# Histone *H3F3A* and *HIST1H3B* K27M mutations define two subgroups of diffuse intrinsic pontine gliomas with different prognosis and phenotypes

**DOI:** 10.1007/s00401-015-1478-0

**Published:** 2015-09-23

**Authors:** David Castel, Cathy Philippe, Raphaël Calmon, Ludivine Le Dret, Nathalène Truffaux, Nathalie Boddaert, Mélanie Pagès, Kathryn R. Taylor, Patrick Saulnier, Ludovic Lacroix, Alan Mackay, Chris Jones, Christian Sainte-Rose, Thomas Blauwblomme, Felipe Andreiuolo, Stephanie Puget, Jacques Grill, Pascale Varlet, Marie-Anne Debily

**Affiliations:** UMR8203 “Vectorologie et Thérapeutiques Anticancéreuses”, CNRS, Gustave Roussy, Univ. Paris-Sud, Université Paris-Saclay, 94805 Villejuif, France; Département de Cancérologie de l’Enfant et de l’Adolescent, Gustave Roussy, Univ. Paris-Sud, Université Paris-Saclay, 94805 Villejuif, France; Département de Neuroradiologie, INSERM U1000, “Imagerie et Psychiatrie”, Hôpital Necker-Enfants Malades, Université Paris V Descartes, Paris, 75015 France; Divisions of Molecular Pathology and Cancer Therapeutics, The Institute of Cancer Research, Sutton, Surrey SM2 5NG UK; Département de Biologie et de Pathologie Médicale, Laboratoire de Recherche Translationnelle, Gustave Roussy, Univ. Paris-Sud, Université Paris-Saclay, Villejuif, 94805 France; Département de Neurochirurgie Pédiatrique, Hôpital Necker-Enfants Malades, Université Paris V Descartes, Paris, 75015 France; Département de Neuropathologie, Hôpital Sainte-Anne, Université Paris V Descartes, Paris, 75014 France; Département de Biologie, Université Evry Val-d’Essonne, 91037 Evry, France

## Abstract

Diffuse intrinsic pontine glioma (DIPG) is the most severe paediatric solid tumour, with no significant therapeutic progress made in the past 50 years. Recent studies suggest that diffuse midline glioma, *H3*-*K27M* mutant, may comprise more than one biological entity. The aim of the study was to determine the clinical and biological variables that most impact their prognosis. Ninety-one patients with classically defined DIPG underwent a systematic stereotactic biopsy and were included in this observational retrospective study. Histone H3 genes mutations were assessed by immunochemistry and direct sequencing, whilst global gene expression profiling and chromosomal imbalances were determined by microarrays. A full description of the MRI findings at diagnosis and at relapse was integrated with the molecular profiling data and clinical outcome. All DIPG but one were found to harbour either a somatic H3-K27M mutation and/or loss of H3K27 trimethylation. We also discovered a novel K27M mutation in *HIST2H3C*, and a lysine-to-isoleucine substitution (K27I) in *H3F3A*, also creating a loss of trimethylation. Patients with tumours harbouring a K27M mutation in H3.3 (*H3F3A*) did not respond clinically to radiotherapy as well, relapsed significantly earlier and exhibited more metastatic recurrences than those in H3.1 (*HIST1H3B/C*). H3.3-K27M-mutated DIPG have a proneural/oligodendroglial phenotype and a pro-metastatic gene expression signature with *PDGFRA* activation, while H3.1-K27M-mutated tumours exhibit a mesenchymal/astrocytic phenotype and a pro-angiogenic/hypoxic signature supported by expression profiling and radiological findings. H3K27 alterations appear as the founding event in DIPG and the mutations in the two main histone H3 variants drive two distinct oncogenic programmes with potential specific therapeutic targets.

**Electronic supplementary material:**

The online version of this article (doi:10.1007/s00401-015-1478-0) contains supplementary material, which is available to authorized users.

## Introduction

High-grade gliomas are the most common paediatric malignant brain neoplasms and among them diffuse intrinsic pontine glioma (DIPG) is a leading cause of death from solid tumours in children, with no improvement in outcome in decades. The median survival below 1 year does not properly describe the various outcomes encountered clinically, from rapid growth over a few months to more prolonged survival up to 2 years in some cases [58]. Histological grading plays no part in diagnosis nor prognostication [7]. Surgery is not offered due to the infiltrative nature of the neoplasm in a delicate brain structure, and biopsy was abandoned in favour of a clinical and radiological diagnosis only [1]. Imaging parameters, when limited to morphological alterations, have not been associated with survival either [26].

Paediatric high-grade gliomas (pHGG) were considered to mimic their adult counterparts until recent genomic studies unravelled significant differences between tumours arising at different ages [2, 10, 41, 42, 58]. A unique epigenetic reprogramming has recently been suggested in pHGG by the discovery of recurrent mutations in genes encoding histone H3 variants, never described in any other type of cancer of any location or age [50, 59]. These hotspot mutations were used to define distinct epigenetic and biological subgroups of HGG specifically seen in children [53]. Supratentorial HGG have recurrent mutations in *H3F3A* encoding histone H3.3, with G34R/V restricted to hemispheric tumours, and K27M to those occurring in the midline [53]. DIPG exclusively harbours K27M mutations, but in histone H3.1 as well as H3.3 genes [8, 20, 56, 59, 60].

K27M mutations in both genes substitute a key lysine residue on the histone H3 tail for a methionine, and have been shown to exert biochemical inhibition of the Polycomb Repressor Complex 2 (PRC2) resulting in a global loss of trimethylation of lysine 27 on all histones H3 molecules either wild types or mutated [34]. However, a closer examination of the epigenome in mutated cells identified subtler deregulation with focal gains and maintenance of H3K27me3, for which the mechanism remains elusive [3, 11]. Although differing only by five amino acids, these two histone H3 proteins are distinct in terms of expression during the cell cycle, chaperones for incorporation in the nucleosomes, localisation on the genome and presumably physiological functions [23, 54]. Consequently, we sought to elucidate if K27M mutations in the distinct histone H3 variants (i.e. *HIST1H3B* and *H3F3A*) were associated with a specific biology and performed comprehensive histological, radiological, transcriptome and CGH array (aCGH) analyses on an extended cohort of DIPG cases biopsied at diagnosis selected with stringent clinical, radiological and histological integrated criteria.

## Materials and methods

### Patients and tumour samples

Patients were selected based on classical clinical and radiological criteria, i.e. short clinical history (<3 months of symptom duration) and the presence of a pontine tumour infiltrating at least 50 % of the pons (suppl. Table S1; suppl. Fig S1) [58]. All patients underwent systematic stereotactic or surgical biopsy at Necker Hospital (Paris, France). Diagnosis of glial infiltrative non-pilocytic neoplasm was histologically confirmed in all patients and we obtained snap-frozen tumour material from 91 children. A smear of each biopsy was performed before freezing and the presence of tumour cells was assessed before their use for the genomic analyses. Informed consent for the translational research programme was obtained from the parents or guardian according to the IRB approved protocol (number DC-2009-955 for tumour banking). Site of the biopsy (routinely the junction between the pons and the cerebellar peduncle where there was an hypersignal on FLAIR sequences) was checked on the post-biopsy imaging [45].

Biopsies from patients with pHGG in a non-brainstem location (*n* = 93) were obtained during the same period at Necker hospital. Their histone H3.1/H3.3 mutation status was determined by Sanger sequencing.

### Sanger sequencing

Histone H3 genes were analysed by direct sequencing of PCR-amplified products from tumour DNA using primers listed in suppl. Table 2.

### Radiological assessment

Morphological sequences of the MRI were reviewed independently by three clinicians (neurosurgeon, neuroradiologist and oncologist). The following criteria were scored on the diagnostic MRI: location, contrast enhancement, ring contrast enhancement, large area of necrosis, cysts, presence of stripes in the infiltrated brain on T2/FLAIR sequences and tumour size. In case of discrepancy, definitive scoring was obtained by consensus. Response to radiotherapy was judged by the clinical improvement only in patients with stable or decreasing doses of steroids to avoid misinterpretation of the radiology due to pseudoprogression [12]. Disease extension was registered throughout the follow-up (i.e. local only, loco-regional or metastatic). Patients without an MRI in the last 2 months were excluded, considering that data were incomplete.

For the diffusion maps acquired at diagnosis, regions of interest (ROI) were drawn over the T2 hyperintensity corresponding to the tumour in each slice, creating a volume of interest (VOI) carefully avoiding necrotic areas, by an experienced neuroradiologist blind to the clinical data. These VOIs were transferred to the co-registered diffusion maps and every voxel value was individually registered. Histograms were created from the apparent diffusion coefficient (ADC) and distributed diffusion coefficient (DDC) voxel data [32].

The performance in terms of prognostication of the recently published “DIPG survival model” was evaluated in comparison to other biological stratification. This score is based on the assessment of the following parameters: age at diagnosis, interval between first symptoms and diagnosis, presence of a ring enhancement and use of adjuvant chemotherapy in addition to radiotherapy [28].

### Histology, immunohistochemistry (IHC) and FISH analyses

Tumours were histologically classified according to WHO 2007 criteria whenever possible. Emphasis was put on the presence of an oligodendroglial component in the tumour cells (morphology, negativity of the tumour cells for vimentin, positivity for OLIG2), presence of interstitial oedema (semi-quantitative) and the presence or absence of necrosis.

A systematic panel of IHC markers was routinely performed: OLIG2, vimentin, GFAP, p53 (DO-7), PTEN, EGFR and MIB-1 as previously described [46]. Additional stainings were developed to detect the loss of nuclear expression of the trimethylation mark at position K27 of the histone 3 tail (1:1000, polyclonal rabbit antibody, Diagenode, Belgium), the nuclear expression of the K27M form of histone H3 (1:1000, polyclonal rabbit antibody, Millipore, CA) and loss of ATRX nuclear expression (1:200, polyclonal rabbit antibody, Sigma-Aldrich, MO).

*PDGFRA* gene copy number was assessed by fluorescent in situ hybridization (FISH) using *PDGFRA*/*CEN4* Dual Color Probe (Abnova, Tapei, Taiwan) on interphase nuclei. In brief, four-micron sections of tumour were mounted on SuperFrost Plus slides (Erie Scientific CA., Portsmouth, NH) and the probed area determined in accordance with haematoxylin and eosin-stained section. The sections were deparaffinised in xylene, rehydrated through an ethanol series, air-dried and incubated in pre-treatment solution (1 M NaSCN-Tris) at 80 °C for 25 min. Slides were then treated with a 0.01 % pepsin solution (Sigma-Aldrich, Saint Louis, USA) at 37 °C for 8 min. After dehydration, 10 µl of probe mixture was applied to each sample, slides were coverslipped and co-denatured at 75 °C for 5 min and hybridized at 37 °C for 48 h using thermobrite system (Leica Biosystems, Richmond, IL). A post-hybridization wash was performed in 2 × SSC at 73 °C for 2 min. Preparations were dehydrated and counterstained with 4,6-diamidino-phenyl-indole (DAPI). Signals were scored in at least 100 non-overlapping interphase nuclei. *PDGFRA* gene amplification was considered as positive in (A) specimens that have ≥40 % of cells displaying ≥4 copies of the PDGFRA signal, (B) specimens that display *PDGFRA* gene amplification, according to one of the following criteria: (a) a PDGFRA to CEN4 ratio ≥2 over all scored nuclei and calculated using the sum of PDGFRA divided by the sum of CEN4 when mean CEN4 per cell is ≥2 copies; (b) the presence of gene cluster (≥4 spots) in ≥10 % of tumour cells; (c) at least 15 copies of the PDGFRA signals in ≥10 % of tumour cells. Results were recorded using a DM600 imaging fluorescence microscope (Leica Biosystems, Richmond, IL) and digital imaging software (Cytovision, v7.4).

### Genomic and statistical analysis

Gene expression (GE) profiling and comparative genomic hybridization on array (aCGH) were conducted for patients with enough material available of required quality on an Agilent platform. For all statistical analysis, the level of significance was 5 %.

### Gene expression microarrays analysis

The data analysed is the result of the gathering of DIPG samples belonging to three different cohorts of young patients with high-grade glioma. In such experimental design, a well-known undesired bias is the batch effect, which is purely technical. To correct for this effect, we replicated some samples in at least two of three batches and performed the ComBat analysis [29] as recommended in Chen et al. [13]. A normal brainstem sample from commercial source, hybridized on chips in the three batches, was used as a reference to normalize the data for the intensity bias as recommended in Do et al. [17]. The normalization of fluorescence intensities is performed in two steps, inspired by Bolstad et al. [5]. The first one is a loess normalization using the normal brainstem samples as a common baseline array for all arrays in the cohort. The second step is a quantile normalization.

Differential analysis was then performed using a moderated *t* test, comparing mean of log2(intensities) in both H3.1- and H3.3-mutated DIPG samples, implemented in the limma R package. The null hypothesis H0 for each gene is that the mean of log2(intensities) is the same in both H3.1- and H3.3-mutated DIPG samples. The alternative hypothesis H1 for each gene is that they are different. The obtained *p* values were adjusted for multiple testing using the Benjamini–Hochberg procedure. The a priori defined level of significance was 5 % after correction for multiple testing.

### Comparative genomic hybridization array analysis

Log2(ratio) between raw signals of the reference DNA and DIPG DNA was first normalized for the dye and intensity effects and also for the local GC % content, using loess procedure, implemented in the limma R package [51]. Data were then centred according to normalized log2(ratio) distribution values by an in-house script using the EM (expectation maximization) approach and aberrations status calling was automatically performed. Normalized centralized values were then segmented using the Circular Binary Segmentation (CBS) algorithm [39], implemented in the DNAcopy R package. The normal copy number interval [log2(ratio) = 0 which means 2 DNA copies as in the DNA reference] was calculated for each sample by multiplying with a define factor the median absolute deviation (MAD) of the normalized data for each single sample.

### Survival curves comparisons

Survival functions were estimated with the Kaplan–Meier method and all survival function estimate comparisons were performed using a log-rank test. The null hypothesis H0 was that the two considered survival function estimates were the same. The alternative hypothesis H1 was that they were different.

### Multivariate survival analysis

The multivariate survival analysis was conducted on H3.1- or H3.3-mutated patients only. The other patients (wild type or H3.2 mutated) were excluded from this analysis. The pool of initial covariates to include in the Cox model was: (1) H3-variant mutation, (2) presence of metastasis, (3) MRI contrast enhancement, (4) treatment type and (5) DIPG Risk score. We first checked for multicollinearity. H3-variant mutation and presence of metastasis are correlated (cor = 0.32) as well as MRI contrast enhancement and the DIPG Risk score (cor = 0.56). We started the analysis with 4 different pools of covariates to separate the correlated pairs of variables and then performed a backward stepwise variable selection using the pec R package. At the end of the procedure, two models retained no variables while the other retained two (‘H3-variant mutation’ and ‘DIPG Risk score’) and three variables (‘H3-variant mutation’, ‘treatment type’ and ‘MRI contrast enhancement’), respectively. For both models, the coefficient for ‘H3-variant mutation’ was 1.4109 and 1.6389, respectively, while the other coefficients were much lower (DIPG Risk score coef = 0.0873, radiotherapy coef = 0.1101, Tarceva coef = −0.4963, Temodal coef = 0.5126, MRI contrast enhancement coef = 0.6573). The hazard ratios were 3.98 and 5.00, respectively, and the confidence intervals were (0.25; 0.92) and (0.67; 1.93), respectively. The log-likelihood *p* values were 3.39e−5 and 7.69e−5, respectively. The H3-variant mutation is then the most important variable in the multivariate Cox model.

### Distribution analyses (diffusion data and age at diagnosis)

To test if both ADC and DDC values were drawn from the same distribution for H3.1- and H3.3-mutated patients, a Mann–Whitney test was performed in each case. The null hypothesis H0 is that ADC (or DDC, respectively) values are drawn from the same distribution law for both H3.1- and H3.3-mutated patients. The alternative hypothesis H1 is that they follow two different distribution laws.

The same methodology was adopted to compare distributions of age at diagnosis between H3.1- and H3.3-mutated patients.

### Proportions comparisons

To compare sex ratios in H3.1- and H3.3-mutated patients, we performed a Chi-squared proportion test as none of the theoretical headcounts was below 5. The null hypothesis H0 was that the male–female proportions are the same in the two considered groups. The alternative hypothesis H1 was that they are different.

For the response to radiotherapy comparison, there was one theoretical headcount below 5, so we performed a Fisher exact test instead of the Chi-squared proportion test. The null hypothesis H0 was that the proportions of good and bad response to radiotherapy were the same in the two considered groups.

## Results

### Loss of H3K27me3 and histone H3 mutation as hallmarks of DIPG

**Fig. 1:**
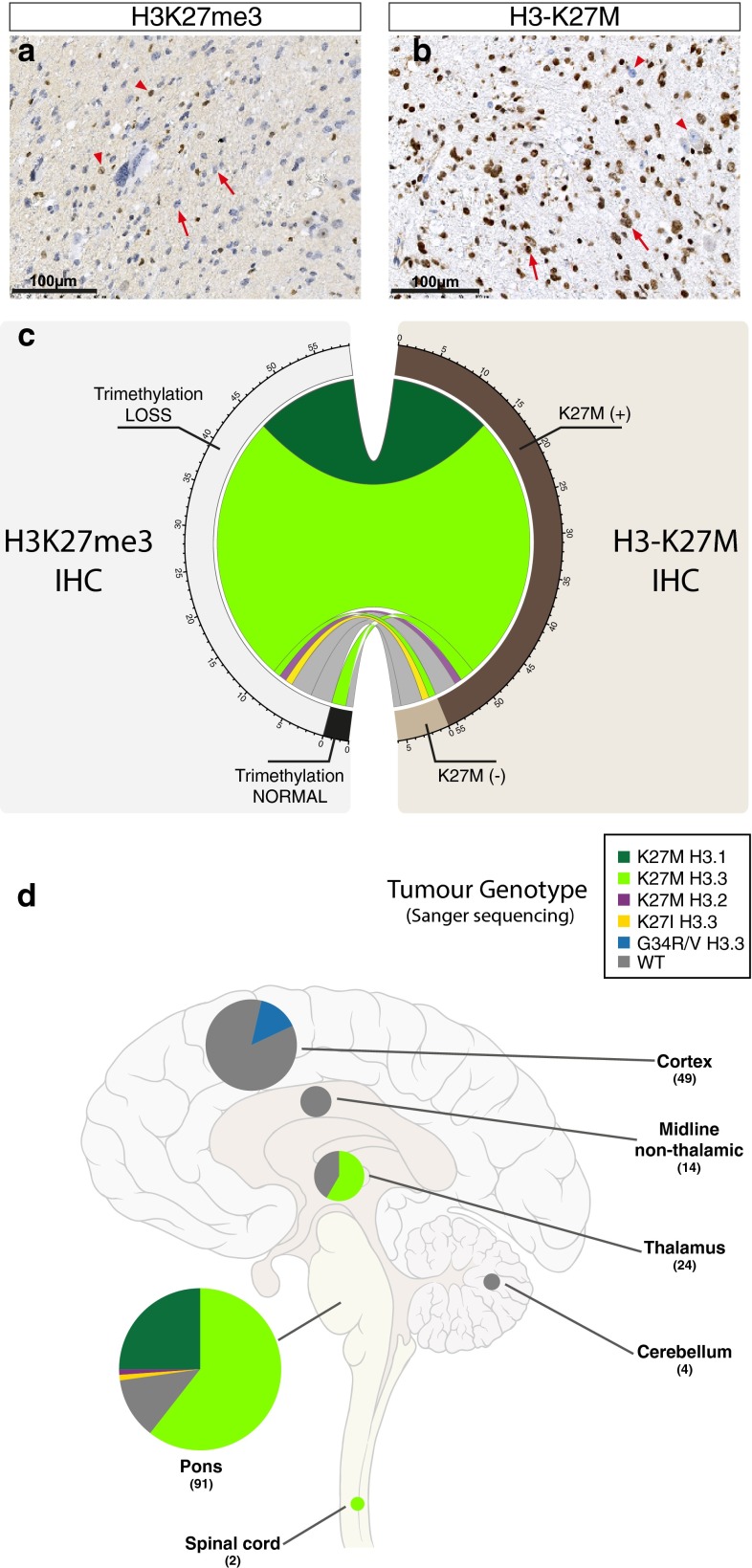
Loss of H3K27me3 as a defining characteristic of DIPG. Representative results of IHC for H3K27me3 (**a**) and H3-K27M (**b**) for a H3.3-K27M-mutated tumour. **a** An overall strong reduction of H3K27me3 is observed with a specific loss of the trimethylation mark in the nuclei of the tumour cells (*arrows*) while the staining is preserved in the nuclei of the normal cells (*triangles*). **b** H3-K27M-mutated proteins are detected in the nuclei of the majority of tumour cells (*brown staining*; *arrows*) with only few normal residual cells merely stained in *blue* (*triangles*) (*scale bar* 100 µm). **c** Plot summarizing the H3K27me3 (*left*) and H3.3-K27M (*right*) immunostaining on 62 DIPG biopsies together with their respective histone mutation status of lysine 27 of H3.1, H3.2 or H3.3 genes indicated by the *colour of the ribbons*. All but one sample showed either H3K27 trimethylation loss or a positive H3.3-K27M immunostaining (61/62; 98 %). The H3-K27M staining allowed detection of the vast majority (>90 %, 56/62) of H3.1- and H3.3-K27M-mutated tumours, and also the rare H3.2-K27M variant. It failed to detect the other rare H3.3-K27I-mutated sample. Overall, 59/62 specimens analysed showed a loss of the trimethylation mark including some histone wild-type (WT) samples (6). **d** Anatomical distribution of histone H3 mutations in 183 pHGG. WT tumours are found throughout the brain (*grey*) whereas H3.3-K27M mutants (**d** *light green*) are found in midline pHGG and H3.3-G34R/V (*blue*) are restricted to the cortex. H3.1-K27M substitutions appeared specific to DIPG tumours and the unique H3.3-K27I and H3.2-K27M found in our cohort also correspond to DIPG

**Fig. 2:**
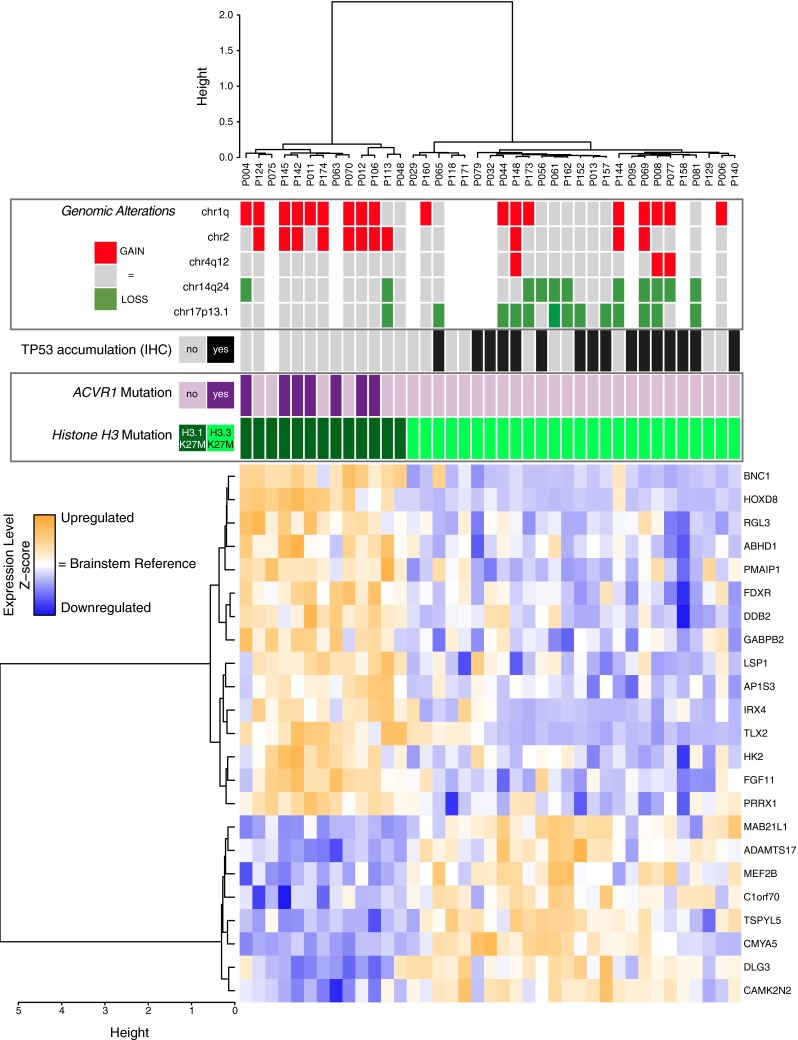
Landscape of genomic alterations in H3.3- and H3.1-mutated tumours. Unsupervised hierarchical clustering and heatmap associated with the gene expression profile of the 23 DEGs (Benjamini and Hochberg adjusted *p* value <0.01) between H3.1- and H3.3-mutated subgroups. The main recurrent genomic alterations found by aCGH, the mutational status of histone *H3F3A* (*light green*), *HIST1H3B* (*dark green*) and *ACVR1* (*purple*) as well as TP53 protein accumulation evaluated by IHC of the 39 primary tumour specimens analysed are reported above the heatmap. TP53 protein accumulation was found exclusively in H3.3 subgroup as well as most genomic imbalances of the corresponding genomic region 17p13.1. Loss of the cytoband 14q24 appears also more often in H3.3 samples whereas H3.1 tumours are characterized by a frequent gain of chromosome 1q and had significantly more aberrations of chromosome 2 (*p* value = 0.0062, Fisher exact test). Gene expression levels in comparison with normal brainstem are illustrated by *varying shades of orange* (upregulation) and *blue* (downregulation)

We analysed a cohort of 62 DIPG biopsy samples obtained at diagnosis for (1) histone H3 lysine 27 trimethylation (Fig. 1a) and (2) immunodetection of the mutated H3-K27M histone (Fig. 1b) by IHC, and correlated these data with the mutational status obtained by Sanger sequencing on corresponding biopsy core (Fig. 1c). We observed an H3K27me3 loss in 95 % of DIPG samples (59/62) (Fig. 1a, c). In the remaining samples, the low tumour infiltration of the assayed sample impeded the detection of the H3K27me3 loss, but H3-K27M-mutated allele could be detected on a distinct sample in two of them, indicating that the lack of IHC detection can result from heterogeneity of distinct tissue sections (Supplemental Figure S2a). For the third patient, although showing typical DIPG MR Imaging, we did not observe a typical histology exhibiting features of a poorly differentiated tumour with pseudopalisading necrosis, mitosis and a giant cell component (P188, suppl. Fig S2b), an absence of OLIG2, but GFAP, S100 and TP53 expression (data not shown). Based on the absence of any H3K27me3 H3-K27M alteration and this particular histology, the sample was not considered as a classical DIPG. Of note, this tumour was not harbouring any IDH1/2 mutation or *MYCN* amplification.

All but one H3-K27M mutation found by sequencing could also be accurately detected by IHC, including a novel mutation a gene encoding the H3.2 variant, *HIST2H3C*, not previously described (Fig. 1c, suppl. Fig S2b, c). However, a second novel mutation, a lysine-to-isoleucine substitution (K27I) in *H3F3A* resulting from two nucleotide changes within the same codon (c.[83A>T; 84G>T]) failed to be detected by the H3-K27M staining but presented a loss H3K27me3 immunoexpression (suppl. Fig S2b, c). In addition, we observed qualitative differences in the labelling intensity of the H3-K27M in the nuclei of the tumour cells between H3.3/H3.2- (strong staining) and H3.1-mutated alleles (weak staining, cross-reactivity corresponding to the important similarity among the two variants already shown [34]) (Fig. 1 a, b, suppl. Fig S2d).

We performed further Sanger sequencing of histones *HIST1H3B*, *H3F3A* and for wild-type cases. we subsequently examined *HIST1H3C* and *HIST2H3C* in an extended cohort of 183 pHGG from diverse anatomical regions. We identified H3.3-K27M mutations in midline tumours whereas H3-G34R/V mutations were restricted to the cerebral hemispheres. Conversely, H3.1- and H3.2-K27M, as well as H3.3 K27I, were only found in pontine tumours (Fig. 1d).

### Landscape of genomic alterations in H3.3- and H3.1-mutated DIPG

Given the discrepancies in H3.1 and H3.3 functions and genomic localization, we next explored the differences between H3.3- and H3.1-mutated tumours using integrated DNA copy number and gene expression analysis.

We identified a signature of 23 and 160 differentially expressed genes (DEGs) using adjusted *p* value cutoff of 1 and 5 %, respectively (Fig. 2, suppl. Fig S3a; suppl. Table 3) which allowed a fair discrimination of the 2 subgroups. Among the 23 most DEGs were four homeobox genes *HOXD8*, *IRX4*, *TLX2* and *PRRX1,* as well as three direct TP53 effectors (*DDB2*, *FDXR* and *PMAIP1*) overexpressed in H3.1- vs. H3.3-mutated tumours.

We further conducted genome-wide aCGH analysis to determine the subgroup specificity of DNA copy number alterations. We observed a more frequent gain of chromosomes 1q (83 vs. 44 %; *p* value = 0.035) and 2 (75 vs. 16 %; *p* value = 0.0008) in the H3.1 subgroup, whereas loss of 17p13.1 (8 vs. 48 %; *p* value = 0.0272) was preferentially found in H3.3 tumours (Fig. 2). These data were significantly correlated with gene expression modulations of the genes located in these regions (data not shown). As the latter locus corresponds to *TP53*, previously shown mutated at least in 40 % of DIPG [8, 20, 24, 60], we evaluated expression by IHC and found exclusive p53 accumulation in H3.3-K27M samples (Fig. 2, S3b; *p* value = 0.0001). Gene set enrichment analysis (GSEA) of our expression profiling data accordingly showed downregulation of TP53 targets (suppl. Fig S3b). Likewise, gain or amplification of *PDGFRA* locus (4q12) was only seen in H3.3 tumours and the overexpression of genes upregulated in *PDGFRA*-amplified pHGG [41] was only observed in the H3.3-K27M subgroup (suppl. Fig S3c, d). ATRX expression was also evaluated by IHC, and ATRX loss was only found in 24 % of H3.3-mutated tumours (suppl. Fig S3e, f). Finally, *ACVR1* mutations were exclusively found in H3.1-K27M tumours (Fig. 2, suppl. Fig S3g) as previously described [8, 20, 56, 60].

### K27M mutations in H3.3 and H3.1 mutations drive two distinct oncogenic programmes

**Fig. 3:**
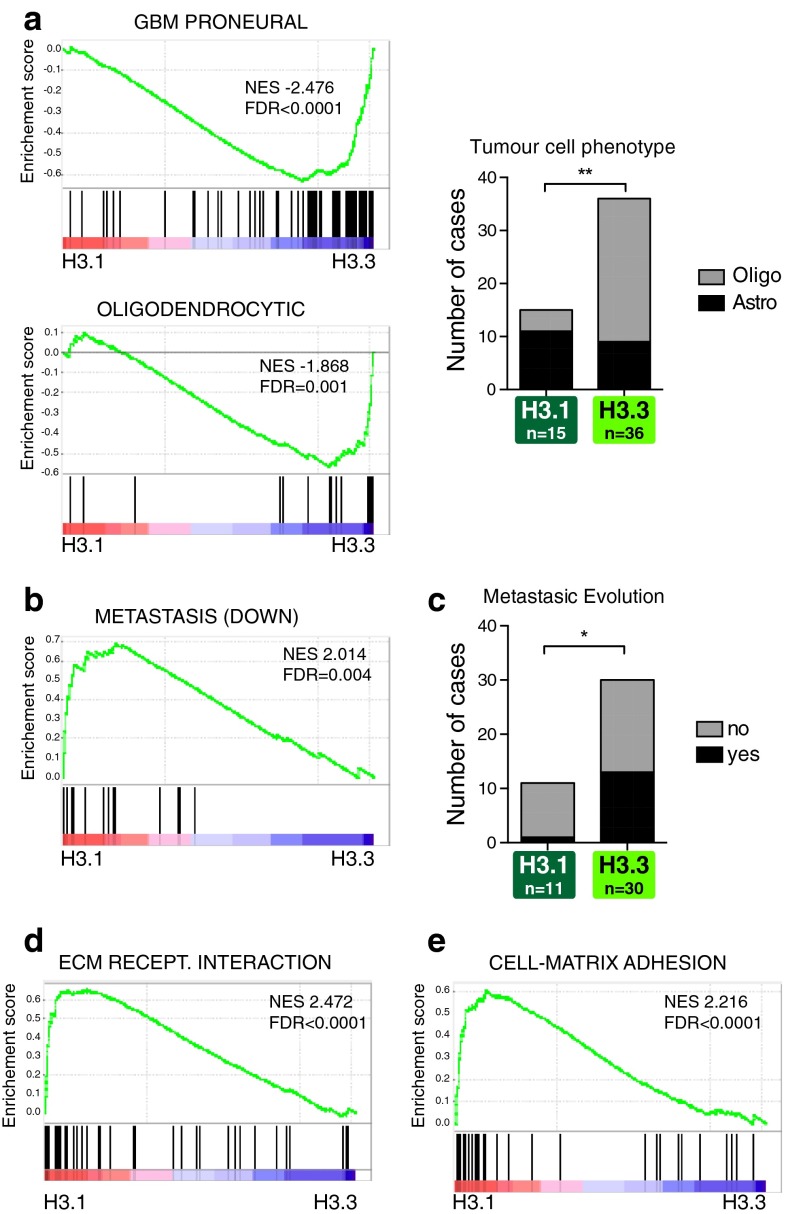
Integrated molecular and phenotypic portrait of H3.3 mutant DIPG. **a** Oligodendrocytic and proneural gene expression signatures. Gene Set Enrichment Analysis (GSEA) was conducted to identify gene sets that exhibited significant overlaps with gene overexpressed in the H3.1-mutated subgroup. The rank order of genes from the most upregulated (*left*, position 1) to the most downregulated (*right*, position 15,198) in H3.1 vs. H3.3 is shown in *x-axis* and the barcode illustrates the position of genes belonging to a particular gene set. The running enrichment score, generated by the cumulative tally of the gene set plotted as a function of the position within the ranked list of array probes, is shown as a *green line*. The total height of the curve indicates the extent of enrichment, with corresponding normalized enrichment score (NES) and false discovery rates (FDR) indicated. Upregulated genes in oligodendrocytes [9] and genes correlated with proneural type of GBM tumours [57] are enriched in H3.3 samples. Accordingly, histology and IHC showed a strong enrichment of tumour with an oligodendrocytic phenotype in H3.3-mutated samples (*right panel*, *p* value = 0.0019; Fisher exact test). **b** Preferential metastatic progression. GSEA analysis shows that the top genes inhibited in metastatic vs. non-metastatic bladder cancer cell lines [22] were also downregulated in H3.3 vs. H3.1 subgroups. **c** Complete clinical follow-up of 41 DIPG patients who had an MRI within the last 2 months of life shows that metastatic evolution was mostly observed in patients with H3.3-K27M mutation (*p* value = 0.04; two-sided Fisher exact test). **d**, **e** Enrichment of genes associated with ‘extracellular matrix receptor interaction’ (KEGG pathway) and ‘cell–matrix adhesion pathways’ (GO:0007160) in H3.3 vs. H3.1 mutant DIPGs

In-depth analysis of GE profiling of the two subtypes showed a strong enrichment for the proneural-glioblastoma multiforme (GBM) [57], oligodendrocytic [9] or neural [21] signatures in H3.3-K27M tumours (Figs. 3a, S4a). With respect to histology, an oligodendroglial differentiation was observed more frequently in H3.3-K27M DIPG (Fig. 3a, *p* value = 0.0019). Moreover, we found a significant downregulation of genes inhibited in metastases [4, 14, 22, 38, 44, 47, 55] (Fig. 3b, suppl. Fig S4b–g). This observation is fully concordant with our clinico-radiological follow-up of 41 DIPG patients who had an MRI within the last 2 months of life, which indicated that all but one of fifteen patients presenting a metastatic relapse belonged to the *H3F3A*-mutated subgroup (*p* value = 0.04). Ten of them had symptoms before death related to the metastases (seizures, raised intracranial pressure without hydrocephalus) (Fig. 3c, suppl. Fig S4 h–p). Related to this, GSEA analysis revealed a depletion for GO categories related to ECM receptor interaction and cell–matrix adhesion—confirming an alteration of adhesion properties in H3.3 tumours (Fig. 3d, e), and also deregulation in gated channel activity-related genes which can modify migration and invasion properties [16, 25] (suppl. Fig S4q).

**Fig. 4:**
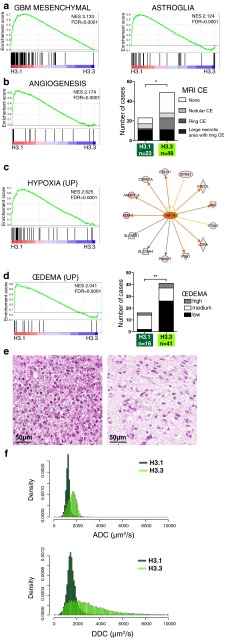
Integrated molecular and phenotypic portrait of H3.1 mutant DIPG. **a** Statistical significant enrichment of mesenchymal-like glioblastoma multiforme (GBM) [57] and astroglia [9] signatures was found in H3.1 tumours by GSEA analyses. Normalized enrichment score (NES) and false discovery rates (FDR) values are indicated. **b** A substantial overrepresentation of genes involved in angiogenesis pathway (biological process GO: 0001525) was identified in H3.1-mutated tumours (*left panel*). This was further confirmed by MRI data (*right panel*) displaying the percentage of samples within H3.1 or H3.3 subgroups associated with distinct contrast enhancement patterns as described in suppl. Fig S6. The distribution of samples within the four classes of patterns is significantly different between H3.1 (*n* = 23) and H3.3 (*n* = 49) subgroups (*p* value = 0.0234, Chi-square test) with a larger proportion of H3.1 samples presenting a large necrotic area. (*CE* contrast enhancement). **c** Accordingly, hypoxic characteristics of H3.1 tumours was revealed by a significant enrichment of the set of genes upregulated by hypoxia in both astrocytes and HeLa cells (*left panel*) [37]. An ingenuity pathway analysis identified HIF1A as a key upstream regulator (activation *z* score = 2.177; *p* value = 7.67e−07) that connects a set of overexpressed genes (*red nodes*) in H3.1 vs. H3.3 samples identified by microarray analysis (*right panel*: *orange edge* leads to activation, *yellow edge* inconsistent state, *grey edge* effect not predicted). **d** Oedema is associated with H3.1 tumours. GSEA analysis shows a significant enrichment of genes upregulated in GBM with oedema (as defined by high FLAIR MRI) for H3.1 vs. H3.3 tumours [61] (*left panel*). Histological analysis of 57 DIPG showed more extensive extracellular oedema in H3.1 than H3.3 tumours (*right panel*; *p* value = 0.0017, Chi-square test). **e** Representative pictures of the histological examination of extracellular oedema in H3.3 (*left panel* sample P044)- and in H3.1-mutated tumour (*right panel* sample P070). Oedema appears at the histological level in H3.1 sample as multiple interstitial clear vacuoles in H&E staining (*scale bar* 50 µm). **f** Voxel-based analyses of apparent diffusion coefficient (ADC, *top panel*) and distributed diffusion coefficient (DCC, *bottom panel*) in 10 H3.1- and 10 H3.3-mutated DIPG. *x-axis* represents the ADC or DDC values in µm^2^/s while the *y-axis* represents the number of pixels with the corresponding value normalized by the total number of pixels analysed (density). ADC and DDC values are overall lower and the spread of the distribution smaller in H3.1 tumours compared to H3.3 ones. Median ADC values are 1228 and 1612 µm^2^/s in H3.1 and H3.3, respectively (*p* < 0.001, Mann–Whitney test) whereas normal brain values are 1000 µm^2^/s. Median DDC values are of 2688 and 1499 µm^2^/s (*p* < 0.001, Mann–Whitney test) in H3.3 tumours compared to H3.1 tumours)

In H3.1-mutated tumours, we identified the overexpression of genes linked to the mesenchymal glioblastoma subtype [21, 57] and astroglial cells [9] (Figs. 4a, S5a). These samples also showed an upregulation of genes involved in angiogenesis and a hypoxia signature with a predicted underlying HIF1A activation (Figs. 4b, c, S5b, c). These molecular characteristics were both corroborated by T1-contrast enhancement MRI data, showing the presence of large necrotic areas in the H3.1-K27M tumours (Figs. 4b, S6). In addition, extracellular oedema appeared as a hallmark of the H3.1 subgroup. First, expression microarray analysis showed a significant enrichment of genes upregulated in GBM with oedema (High Flair; Fig. 4d). In addition, histological analysis of our extended cohort of 57 DIPG showed more extensive extracellular oedema in H3.1 than H3.3 tumours with the presence of extracellular vacuoles (Fig. 4d,e, *p* value 0.0017). Finally in the diffusion-weighted MR imaging, H3.1- and H3.3-mutated tumours exhibited different distribution of the ADC and more strikingly DDC values, pointing to differential water distribution (Fig. 4f).

### *HIST1H3B* K27M mutation is associated with a less aggressive behaviour in DIPG

**Fig. 5:**
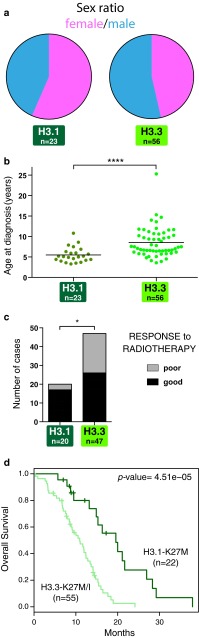
Histone H3 mutations define two clinical DIPG subgroups. **a** Sex distributions of cases with H3.1 or H3.3 mutations show a trend towards an increased proportion of females in H3.1 mutant samples (56.5 vs. 46.4 %) which did not reach statistical significance. **b** Age distribution of H3.1 and H3.3 patients at diagnosis. The median age at diagnosis was significantly smaller in H3.1 (5.1 years) in comparison with H3.3 (7.4 years). Statistical significance was calculated with Mann–Whitney test (median, ****p value <0.0001, *n* = 79). **c** Bar diagrams representing the response to radiation therapy stratified by H3.1 and H3.3 mutation status. The vast majority of H3.1-mutated patients (85 %) were identified as good responders to radiation therapy whereas H3.3-mutated cases are divided evenly between the responders and non-responders group (55.3 %, *p* value = 0.0263, two-sided fisher exact test). **d** Kaplan–Meier estimates of the survival stratified by H3.1- or H3.3-K27M mutations. H3.1 tumours are associated with a better overall survival than H3.3 tumours (median survival times of 15.0 and 9.2 months, respectively, *p* value = 4.51e−05; log-rank test)

Given the aforementioned differences in H3.1- and H3.3-K27M tumours, we next compared their clinical characteristics. We did not find any significant difference in terms of sex ratio (Fig. 5a), but found an earlier onset of the disease for H3.1 patients of approximately 2 years (Fig. 5b; *p* value <0.0001, Mann–Whitney test) in our cohort and in a previously published one [8, 20, 60] (suppl. Fig S7a, b). More importantly, H3.1 patients were associated with a better clinical response to radiotherapy (85 vs. 55.3 % of good clinical responders, *p* value = 0.0263), and overall survival length (OS), with a median OS of 15.0 months for patients with H3.1 mutations compared to 9.2 months for H3.3-mutated patients (Fig. 5c, d; *p* value = 4.51e−05, log-rank test). Re-analysis of published cases confirmed the overall better prognosis of H3.1-K27M tumours in an independent cohort [60] (suppl. Fig S7c). Finally, we tested in our cohort other previously published prognostic classifications based on *ACVR1* mutation (suppl. Fig S7d, e), Histone H3 WT/K27M status [31] (suppl. Fig S7f), or stratification according to metastatic evolution or treatment administered (suppl. Fig S7g, h), clinico-radiological risk score [28] (suppl. Fig S7i), MRI contrast enhancement [43] (suppl. Fig S7j). None of these risk factors was a stronger predictor for survival than the type of mutated histone H3 which remained as such in multivariate analysis (*p* value <0.0001).

## Discussion

Mutations in the N-terminal tail of histone H3 genes have been recently found in midline pHGG and at particular high frequency in DIPG. Our results support that substitution of the lysine at position 27 of genes encoding H3, which can be detected by IHC through its consequences, i.e. the global loss of trimethylation on this residue, is the driving event in DIPG oncogenesis. In addition to K27M mutations in genes encoding H3.3 (*H3F3A*) and H3.1 (*HIST1H3B* and *HIST1H3C*) found in the majority of samples as previously reported, we also identified two novel lysine 27 substitutions. The first was a novel K27M mutation in a gene encoding histone H3.2 (*HIST2H3C*) implying that whichever histone H3 variant is targeted by the mutation (H3.1, H3.2 or H3.3), it may lead to the development of a DIPG. A further argument for the importance of alterations of these residues is the discovery of a second novel mutation, two base changes being required to produce a K27I lysine-to-isoleucine substitution in H3.3 (*H3F3A*). Although reported here in a DIPG sample for the first time, systematic in vitro modification of the lysine 27 predicted K27I as the only substitution other than K27M to result in a repressive effect on H3K27me3 [34]. In summary, our data underline the pivotal importance in this disease of H3K27 mutations and loss of H3K27me3, which is consequently characteristic of DIPG and likely represents the first genomic event leading to transformation in these tumours. DIPG thus appears as a homogeneous tumour group defined as a glial neoplasm with a stereotypical epigenetic drive consecutive to these histone H3 mutations.

All H3K27 mutations described in DIPG appear to have the same epigenomic consequences on the PRC2 complex in general [11, 34] despite the distinct functions and genomic distribution of the different variants. Critically, it is clear that the type of histone targeted by K27 alterations greatly influences the survival length of patients. H3.1-mutated tumours were found to respond better to the treatment, mainly radiotherapy, have a less aggressive course and metastasize less frequently. As the significant effect on OS length was confirmed by the re-analysis of an independent series [60], assessment of the type of histone mutation could be used as a prognostic stratification factor in future prospective studies. Moreover, in multivariate analysis considering also the effect of treatment, the type of histone H3 mutated was a better predictor for survival length than the DIPG clinico-radiological risk score [28] or the presence of an *ACVR1* mutation identified in a subset of DIPG with a less aggressive course [56, 60]. The latter results concerning *ACVR1* mutation can be explained by the segregation of this alteration with H3.1-K27M mutation rather than the reverse. Indeed, no difference was observed among the H3.1 tumour subgroup between *ACVR1* WT- and *ACVR1*-mutated samples (Fig S7e). Also, the high frequency of metastatic progression in *H3F3-*mutated DIPG, if confirmed in an independent series, could suggest a different therapeutic approach in the subset of patients with tumours prone to dissemination.

Our GE profiling data suggest that the two main histone H3 mutations drive distinct oncogenic programmes, an observation further strengthened by the integration of histological and MR-imaging data. Notably, the less aggressive H3.1 tumours appeared to be more hypoxic and oedematous, as well as exhibiting higher expression of neo-angiogenic markers than their H3.3 counterpart. A bias towards oligodendroglial and astroglial differentiation was observed for H3.3 and H3.1 tumours, respectively. Also, the dramatic metastatic evolution of the disease—observed almost exclusively in H3.3-K27M samples, could be partially explained by the identification of deregulated genes encoding ion-gated channels and adhesion molecules known to play a role in this invasion process [15, 52]. Despite their different progression rate, we did not find discrepancies in MIB-1 proliferative index between the two groups.

We show here that the H3-K27M-mutated DIPG group recognized by previous studies [7, 8, 31] could be further divided into two subgroups, H3.1-K27M and H3.3-K27M. There is significant overlap between the H3.3-K27M subgroup and the PDGFRA-driven DIPG shown previously and between the H3.1-K27M subgroup and the tumours with a mesenchymal gene expression signature [40, 46]. The wild-type DIPG (with respect to histone H3 mutation) described by other groups [8, 31], is also found in our series, but we did not find differences in survival between WT and H3-K27-mutated groups in this population of strictly defined DIPG (suppl. Fig S7f). Importantly, all but one of the silent tumours we describe had a loss of the trimethylation mark at K27 of the histone H3. As this is an indication of the functional impairment of the PRC2 complex, alternative mechanisms have to be explored to confirm these tumours could be grouped together with the histone H3-27 M-mutated ones. We did not find a *MYCN* amplification, nor an *IDH1/2* mutation in the only sample without trimethylation loss at K27 of the histone H3 (case P188; suppl. Fig S2). *MYCN*-amplified cases may correspond to a PNET-like variant and are in fact relatively rare (less than ten cases reported so far in the whole literature with *MYCN* amplification).

The differences between the H3.3/H3.1 subgroups may be a result of distinct cells of origin for these tumours, or reflect a mechanistic consequence of the type of histone mutated. Interestingly, we identified a differential expression between these subgroups of numerous homeobox genes, known to be pivotal for neural stem cell identity and tissue patterning. This coupled with the earlier age of onset of the H3.1-mutated tumours as well as their restricted pontine location (as opposed to H3.3 mutations also found in HGG of other midline regions) could imply that distinct progenitors are targeted by the two mutations. Equally, homeobox clusters are known to be regulated by the PRC2 complex [49] and the epigenetic consequences of the PRC2 complex inhibition might have regional differences depending on the histone type. Secondary genetic alterations also co-segregate between the two subgroups. *PDGFRA* amplification/mutation, a known inducer of oligodendrocytic differentiation [6, 19, 48], is seen together only with histone H3.3 mutations, whilst *ACVR1* mutations that activate the BMP pathway driving astrogenesis are only concomitantly observed with mutant H3.1. Still, the gene expression signature between H3.1 and H3.3 subgroups may not be due to the H3 mutations themselves but rather the accompanying alterations (*PDGFRA* vs. *ACVR1*) or differential modification of the microenvironment by tumour cells. However, the astrocytic phenotype was observed in the H3.1-mutated tumours independently of the *ACVR1* mutation and future work in vitro should help unravel this matter.

In conclusion, biopsies performed at diagnosis allowed us to confirm a DIPG in almost every case by the identification of a specific histone H3K27 alteration. The type of histone H3 mutated could also predict the outcome of DIPG patients more efficiently than clinical and radiological characteristics of the tumours. Moreover, two distinct oncogenic pathways associated with the mutated histone-driven subgroups have been identified in DIPG and different therapeutic approaches may be developed to target these specific alterations and phenotypic changes. Conversely, mutually exclusive alterations observed in the two DIPG subgroups may influence the response to a given targeted agent. Crucially, our results justify the re-introduction of stereotactic biopsies at diagnosis in the management of DIPG for better treatment stratification decisions.

## Electronic supplementary material

Supplementary material 1: **Figure S1** Flow diagram of patient selection process. Flow diagram describing the patient selection process and exclusion reasons for each statistical analysis. In bold frames, the patient counts, the grey backgrounds denote the statistical analysis and the dashed frames the exclusion criteria. (PDF 213 kb)

Supplementary material 2: **Figure S2** General and specific features of mutant H3K27 and H3K27me3 immunohistochemistry in DIPG. (a) H3-K27M IHC on an entire biopsy core from a H3.3 mutated DIPG (Scale bar: 1 mm). An important heterogeneity in H3-K27M detection can be observed along the same biopsy as shown by the important density of nuclei stained on the right side of the sample –reflecting an important infiltration of cancer cells, as opposed to the left side where isolated tumour cells can still be identified with the antibody against the mutant H3.3 protein. (b) Immunohistochemistry for H3K27me3 and H3-K27M as well as H&E staining are shown for the 3 atypical samples: P198, H3.2-K27M (Scale bar: 100 µm), P180, H3.3-K27I (Scale bar: 100 µm) and P188, WT DIPG samples (Scale bar: 50 µm). Both H3.2-K27M and H3.3-K27I samples are associated with a global loss of the trimethylation mark in a similar way to H3.1- and H3.3-K27M tumours. The H3.2 specimen is strongly stained by the antibody specific of K27M proteins as H3.3 tumours, unlike H3.3-K27I for which no staining was observed. In the P188 case, neither a trimethylation loss, nor a K27M staining was observed.. The histology of this sample exhibit features of a poorly differentiated GBM with pseudopalisading necrosis, mitosis and a giant cell component as seen on the H&E staining. (c) H3.2-K27M and H3.3-K27I somatic mutations. Sanger sequencing chromatograms showing the *H3F3A* double mutation encoding a p.K27I substitution and the *HIST2H3C* mutation encoding a p.K27M substitution in the indicated DIPG cases. Mutation positions are shown compared to matched normal DNA and indicated in yellow. (d) Representative results of IHC for H3K27me3 and H3-K27M for a H3.1-K27M mutated tumour biopsy. (Left panel) A strong reduction of overall H3K27me3 is observed with a specific loss of the trimethylation mark in tumour glial cells as revealed by nuclei blue counterstaining (arrows) where the nuclei of normal cells remain labelled in brown (i.e. for example capillary endothelial cells; triangles). (Right panel) H3-K27M mutated proteins are detected in the nuclei of the majority of the infiltrative tumour cells (brown staining; arrows) with only few normal residual cells merely stained in blue (triangles). The mutated protein nuclear staining is reproducibly fainter as compared to H3.3 mutated tumours (for comparison see Fig. 1A.) (Scale bar: 50 µm). (PDF 3373 kb)

Supplementary material 3. **Figure S3A** Molecular signature of H3.1 and H3.1 subgroups. (a) Unsupervised hierarchical clustering and heatmap associated with the gene expression profile of the 160 differentially expressed genes (Benjamini & Hochberg adjusted *p*-value < 0.05) between H3.1 and H3.3 subgroups. The mutational status of histone *H3F3A* (light green), *HIST1H3B* (dark green) or WT for those two genes as well as *HIST2H3C (*grey) are reported above the heatmap. The affiliation (plain colour) or similarity (light colour) of each sample to the two K-means subgroups identified in a previous study are indicated in blue for km1 and purple for km2 [46]. Overall, H3.1 mutated samples are preferentially associated with a km2 signature (8/13 DIPG samples) whereas H3.3 preferentially show a km1 expression profile (23/26 samples, fisher’s exact test, *p*-value = 0.0021). (PDF 229 kb)

Supplementary material 4: **Figure S3B** Molecular signature of H3.1 and H3.1 subgroups. (b) TP53 protein was assessed by IHC on 57 DIPG biopsies. p53 accumulation was restricted to the H3.3 mutated subgroup (*p*-value = 0.0001; fisher exact test, top panel). A gene signature comprising upregulated targets of TP53 in a cancer cell line strongly enriched among H3.1 tumours [30], pointing to an inactivation of TP53 transcriptional activity in H3.3-mutated samples (bottom panel). (c) Genomic alterations (amplification or gain) of *PDGFRA* identified by aCGH & FISH analyses were found exclusively in H3.3-K27M samples (p-value = 0.428; two-sided fisher exact test, top panel). Genes preferentially upregulated in ‘*PDGFRA*-amplified pHGG’ [41] were significantly overexpressed in H3.3 vs. H3.1 tumours as shown on the GSEA enrichment plot (bottom panel). (d) Representative results of *PDGFRA/CEN4* FISH showing no amplification (top panel) or often innumerable red PDGFRA signals, but only a few green CEN4 signals per nucleus indicating a *PDGFRA* gene amplification (bottom panel; 600X magnification). (e) Bar graphs showing an exclusive loss of ATRX expression in H3.3-K27M tumours (p-value = 0.152, two-sided fisher exact test). (f) Representative results of IHC for ATRX in H3.1/3-K27M mutated tumour biopsies. (Top panel) A strong nuclear staining is observed in all tumour and normal cells. (Bottom panel) A loss of ATRX staining is observed in tumour glial cell nuclei as revealed by nuclei blue counterstaining while the nuclei of normal cells remain labelled in brown (for example endothelial cells). (Scale bar: 100 µm). (g) Bar graphs showing segregation of activating mutations in *ACVR1* with H3.1-K27M mutation (p-value < 0.0001, two-sided fisher exact test) in a series of 53 DIPG cases. (PDF 850 kb)

Supplementary material 5: **Figure S4A** Recurrent molecular alterations in H3.3-K27M DIPG and metastases identified by imaging. (a) GSEA analysis demonstrated that DIPG with H3.3-K27M mutation are significantly enriched in the ‘*neuronal*’ gene set reported in Freije et al. (HC1B gene set) [21]. (b-g) GSEA analysis showed that the top genes inhibited in metastatic vs. non-metastatic head and neck squamous cell carcinoma tumour [47] (b-c), endometrial tumours [4] (d), colorectal carcinoma cells [44] (e), pancreatic cancer cells [38] (f) were also downregulated in H3.3 vs. H3.1 subgroups. Conversely, top genes induced in metastatic breast adenocarcinoma [55] were upregulated in H3.3 vs. H3.1 tumours (g). (PDF 176 kb)

Supplementary material 6: **Figure S4B** Recurrent molecular alterations in H3.3-K27M DIPG and metastases identified by imaging. (h-p) Metastases in H3.3 mutated DIPG were assessed on MRI performed within the last two months prior to death. Two distinct patterns were observed. Firstly, a linear contrast enhancement of the meninges was apparent, indicating either a loco-regional leptomeningeal spread in the cerebellum or in the cerebrum (h, i, j) or a distant leptomeningeal spread in the spinal cord (k, l, m). Second, a subependymal spread forming nodules in the ventricles was visible as hypersignal on diffusion-weighted imaging (n), T1-weighted sequences with gadolinium (o) or FLAIR imaging (p). (q) Gene list functional enrichment using TOPPFun (https://toppgene.cchmc.org/) of the list of 160 differentially expressed genes between the two tumour subgroups highlighted *‘gated channel activity’* (GO:0022836, *p*-value = 1.06 e-6 and Benjamini & Hochberg FDR = 2.893 e-4) and *‘ion channel activity’* (GO:0005216, *p*-value = 1.101 e-5 and Benjamini & Hochberg FDR = 1.567 e-3) molecular functions as well as *‘cation channel complex’* cellular component (GO:0005261, *p*-value = 3.413 e-6 and Benjamini & Hochberg FDR = 4.659 e-4) as significantly enriched. The majority of the genes involved in those categories are downregulated in H3.1 vs. H3.3 samples as reflected by their fold change and corresponding *p*-value. (PDF 716 kb)

Supplementary material 7: **Figure S5** Recurrent molecular alterations in H3.1-K27M DIPG. (a) GSEA analysis demonstrated that upregulated genes in DIPG with H3.1-K27M mutation are significantly enriched in the ‘mesenchymal’ gene set reported in Freije et al. (HC2B gene set) [21]. (b) Hypoxic characteristic of H3.1 tumours was revealed by a significant enrichment of several hypoxia-related gene sets reinforcing the results presented in Fig. 3b, representing upregulated genes under hypoxic conditions or after HIF1A overexpression [18, 27, 30, 33, 36] as well as the ‘*response to hypoxia*’ biological process (GO:0001666) and ‘*Biocarta P53 hypoxia’* pathway. (c) In addition to the angiogenesis pathway both ‘*vasculature development’* biological process (GO:0001944) and genes identified in processes related to *‘tumour vascularization’* [35] were found enriched in upregulated DEGs in H3.1-mutated samples. The normalized enrichment score (NES) and false discovery rate (FDR)-corrected q-value are indicated. (PDF 196 kb)

Supplementary material 8: **Figure S6** Radiological classification of neoangiogenesis and necrosis. Contrast enhancement indicative of neoangiogenesis and necrosis were graded on T1 + contrast MRI sequences acquired on the axial plane for each patient and indicated by an arrow on the left side and schematized on the right. Images are displayed that represent each configuration in terms of (a) absence of contrast, (b) nodular contrast enhancement, (c) ring contrast enhancement, (d) large necrotic area with ring enhancement. (PDF 449 kb)

Supplementary material 9: **Figure S7** In-depth analysis of clinical parameters differentiating DIPG subgroups. (a) Sex distribution of cases with H3.1 and H3.3 mutations in a cohort of 92 DIPG from published datasets [8, 20, 60]. No difference of sex proportion was observed. (b) Age distribution of H3.1 and H3.3 patients at diagnosis. The median age at diagnosis was significantly smaller in H3.1- (4.4 years, *n* = 21) in comparison with H3.3-mutated samples (7.0 years, *n* = 71) from published studies [8, 20, 60] as observed in our cohort (Fig. 5b). Statistical significance was calculated with Mann–Whitney test (median, ****p-value < 0.0001). (c) Kaplan–Meier estimates of the survival stratified by H3.1 or H3.3-K27M mutations in the published data from Wu et al. [60]. H3.1 tumours (*n* = 12) are associated with a better overall survival than H3.3 tumours (*n* = 31) (median survival times of 14.29 and 8.74 months, respectively; *p*-value = 0.0351; log-rank test). (d-e) Overall survival curves of all *ACVR1* mutated or *ACVR1* WT tumours in our cohort highlight an increased median survival time for mutated samples (d, median survival times of 16.21 and 9.71 months respectively; *p*-value = 0.00496; log-rank test). However, no difference was observed among the H3.1 tumour subgroup between *ACVR1* WT and *ACVR1* mutated samples (e*, p*-value = 0.845; log-rank test, data compiled from our cohort with a previously published one [60] ). (f) Overall survival curves of all DIPG from the cohort stratified by *H3*-*K27M* mutation or *WT* status. No difference in outcome was associated with *H3*-*K27M* tumours (*n* = 78) vs. the *WT* tumours (*n* = 11) (*p*-value = 0.114; log-rank test). (g) Influence of a metastatic evolution on DIPG overall survival curves. A shorter overall survival was observed for patients presenting metastasis development during the course of the disease (*n* = 16) compared to the others (*n* = 30, *p*-value = 0.0357; log-rank test). (h) Overall survival curves of patients with DIPG stratified according to the treatment. Clinical outcome was comparable for patients with treated by radiotherapy (RT), RT + Tarceva, RT + Temodal or other treatments (*p*-value = 0.407; log-rank test), indicating that no bias was introduced in our survival data by the treatment effect. (i) Stratification of our DIPG samples according to the DIPG clinico-radiological risk score. Overall survival curves of patients with tumours belonging to the three distinct classes of DIPG defined by the predictor developed by Jansen et al. were generated [28]. The computation of the DIPG risk score is based on several clinical parameters (age at diagnosis, ring enhancement, months of symptom duration, the use of oral/intensive chemotherapy in addition to RT). No significant difference was observed between the three classes of standard, intermediate and high risk (*p*-value = 0.101; log-rank test) even if a similar trend to the Jansen’s study was observed with a slightly shorter median survival time for high risk DIPG samples. (j) Stratification of DIPG according to contrast enhancement (MRI). As contrast enhancement has been suggested to correlate with a worse prognosis in some studies [43], we investigated the correlation between contrast enhancement and outcome. There was no significant difference in overall survival according to the presence or absence of contrast enhancement (*p*-value = 0.485). (PDF 232 kb)

Supplementary material 10: **Table S1:** Patient summary data. Clinical, histopathological, radiological and molecular variables in the cohort of 91 DIPG of the study. “FU”: Follow-up in month; “DIPG_survival model”: score according to the model in Jansen et al.; “DIPG survival model 3 classes”: classification into 3 classes of low intermediate and high risk according to Jansen et al.; “Metastasis”: presence or absence of metastases identified by MRI within the last 3 months of life; “Km1/Km2 Group” : belonging to the Km1 or Km2 groups based on gene expression profile as defined in Puget et al; “Tumour Compartment Oligo/Astro” : Oligodendroglial or astrocytic differentiation of the tumour cells; “WHO” : grading according to the WHO classification; “CHSA” : grading according to the Sainte-Anne Hospital classification; “Oedema”: presence of extracellular oedema on histological sections; “Type of Contrast Enhancement (MRI)”: classification in 4 classes of CE as defined in suppl. Fig S6; “NA”: Data not available; “NI”: Non-interpretable. **Table S2:** PCR primers. **Table S3:** List of differentially expressed genes. Genes significantly differentially expressed between H3.1 and H3.3 tumour subgroups as determined by moderated t-test using an adjusted *p*-value cutoff of 5 % are indicated with their corresponding ratio and *p*-value. Genes in bold correspond to those with an adjusted *p*-value cutoff of 1 %. (PDF 167 kb)

## References

[1] Albright AL, Packer RJ, Zimmerman R (1993). Magnetic resonance scans should replace biopsies for the diagnosis of diffuse brain stem gliomas: a report from the Children’s Cancer Group. Neurosurgery.

[2] Bax DA, Mackay A, Little SE (2010). A distinct spectrum of copy number aberrations in pediatric high-grade gliomas. Clin Cancer Res Off J Am Assoc Cancer Res.

[3] Bender S, Tang Y, Lindroth AM (2013). Reduced H3K27me3 and DNA Hypomethylation Are Major Drivers of Gene Expression in K27M Mutant Pediatric High-Grade Gliomas. Cancer Cell.

[4] Bidus MA, Risinger JI, Chandramouli GVR (2006). Prediction of lymph node metastasis in patients with endometrioid endometrial cancer using expression microarray. Clin Cancer Res Off J Am Assoc Cancer Res.

[5] Bolstad BM, Irizarry RA, Astrand M, Speed TP (2003). A comparison of normalization methods for high density oligonucleotide array data based on variance and bias. Bioinforma Oxf Engl.

[6] Bouvier C, Bartoli C, Aguirre-Cruz L (2003). Shared oligodendrocyte lineage gene expression in gliomas and oligodendrocyte progenitor cells. J Neurosurg.

[7] Buczkowicz P, Bartels U, Bouffet E (2014). Histopathological spectrum of paediatric diffuse intrinsic pontine glioma: diagnostic and therapeutic implications. Acta Neuropathol (Berl).

[8] Buczkowicz P, Hoeman C, Rakopoulos P (2014). Genomic analysis of diffuse intrinsic pontine gliomas identifies three molecular subgroups and recurrent activating ACVR1 mutations. Nat Genet.

[9] Cahoy JD, Emery B, Kaushal A (2008). A transcriptome database for astrocytes, neurons, and oligodendrocytes: a new resource for understanding brain development and function. J Neurosci.

[10] De Carli E, Wang X, Puget S (2009). IDH1 and IDH2 mutations in gliomas. N Engl J Med.

[11] Chan K-M, Fang D, Gan H (2013). The histone H3.3K27M mutation in pediatric glioma reprograms H3K27 methylation and gene expression. Genes Dev.

[12] Chassot A, Canale S, Varlet P (2012). Radiotherapy with concurrent and adjuvant temozolomide in children with newly diagnosed diffuse intrinsic pontine glioma. J Neurooncol.

[13] Chen C, Grennan K, Badner J (2011). Removing batch effects in analysis of expression microarray data: an evaluation of six batch adjustment methods. PLoS One.

[14] Cromer A, Carles A, Millon R (2004). Identification of genes associated with tumorigenesis and metastatic potential of hypopharyngeal cancer by microarray analysis. Oncogene.

[15] Cuddapah VA, Robel S, Watkins S, Sontheimer H (2014). A neurocentric perspective on glioma invasion. Nat Rev Neurosci.

[16] Cuddapah VA, Sontheimer H (2011). Ion channels and transporters [corrected] in cancer. 2. Ion channels and the control of cancer cell migration. Am J Physiol Cell Physiol.

[17] Do JH, Choi D (2006). Normalization of microarray data: single-labeled and dual-labeled arrays. Mol Cells.

[18] Elvidge GP, Glenny L, Appelhoff RJ (2006). Concordant regulation of gene expression by hypoxia and 2-oxoglutarate-dependent dioxygenase inhibition: the role of HIF-1alpha, HIF-2alpha, and other pathways. J Biol Chem.

[19] Finzsch M, Stolt CC, Lommes P, Wegner M (2008). Sox9 and Sox10 influence survival and migration of oligodendrocyte precursors in the spinal cord by regulating PDGF receptor alpha expression. Dev Camb Engl.

[20] Fontebasso AM, Papillon-Cavanagh S, Schwartzentruber J (2014). Recurrent somatic mutations in ACVR1 in pediatric midline high-grade astrocytoma. Nat Genet.

[21] Freije WA, Castro-Vargas FE, Fang Z (2004). Gene expression profiling of gliomas strongly predicts survival. Cancer Res.

[22] Gildea JJ, Seraj MJ, Oxford G (2002). RhoGDI2 is an invasion and metastasis suppressor gene in human cancer. Cancer Res.

[23] Goldberg AD, Banaszynski LA, Noh K-M (2010). Distinct factors control histone variant H3.3 localization at specific genomic regions. Cell.

[24] Grill J, Puget S, Andreiuolo F (2012). Critical oncogenic mutations in newly diagnosed pediatric diffuse intrinsic pontine glioma. Pediatr Blood Cancer.

[25] Haas BR, Sontheimer H (2010). Inhibition of the sodium–potassium–chloride cotransporter isoform-1 reduces glioma invasion. Cancer Res.

[26] Hargrave D, Chuang N, Bouffet E (2008). Conventional MRI cannot predict survival in childhood diffuse intrinsic pontine glioma. J Neurooncol.

[27] Harris AL (2002). Hypoxia–a key regulatory factor in tumour growth. Nat Rev Cancer.

[28] Jansen MH, Veldhuijzen van Zanten SE, Sanchez Aliaga E (2015). Survival prediction model of children with diffuse intrinsic pontine glioma based on clinical and radiological criteria. Neurooncol.

[29] Johnson WE, Li C, Rabinovic A (2007). Adjusting batch effects in microarray expression data using empirical Bayes methods. Biostat Oxf Engl.

[30] Kannan K, Amariglio N, Rechavi G (2001). DNA microarrays identification of primary and secondary target genes regulated by p53. Oncogene.

[31] Khuong-Quang D-A, Buczkowicz P, Rakopoulos P (2012). K27M mutation in histone H3.3 defines clinically and biologically distinct subgroups of pediatric diffuse intrinsic pontine gliomas. Acta Neuropathol (Berl).

[32] Kwee TC, Galbán CJ, Tsien C (2010). Comparison of apparent diffusion coefficients and distributed diffusion coefficients in high-grade gliomas. J Magn Reson Imaging JMRI.

[33] Leonard MO, Cottell DC, Godson C (2003). The role of HIF-1 alpha in transcriptional regulation of the proximal tubular epithelial cell response to hypoxia. J Biol Chem.

[34] Lewis PW, Müller MM, Koletsky MS (2013). Inhibition of PRC2 activity by a gain-of-function H3 mutation found in pediatric glioblastoma. Science.

[35] Lu C, Bonome T, Li Y (2007). Gene alterations identified by expression profiling in tumor-associated endothelial cells from invasive ovarian carcinoma. Cancer Res.

[36] Manalo DJ, Rowan A, Lavoie T (2005). Transcriptional regulation of vascular endothelial cell responses to hypoxia by HIF-1. Blood.

[37] Mense SM, Sengupta A, Zhou M (2006). Gene expression profiling reveals the profound upregulation of hypoxia-responsive genes in primary human astrocytes. Physiol Genomics.

[38] Nakamura T, Fidler IJ, Coombes KR (2007). Gene expression profile of metastatic human pancreatic cancer cells depends on the organ microenvironment. Cancer Res.

[39] Olshen AB, Venkatraman ES, Lucito R, Wigler M (2004). Circular binary segmentation for the analysis of array-based DNA copy number data. Biostat Oxf Engl.

[40] Paugh BS, Broniscer A, Qu C (2011). Genome-wide analyses identify recurrent amplifications of receptor tyrosine kinases and cell-cycle regulatory genes in diffuse intrinsic pontine glioma. J Clin Oncol Off J Am Soc Clin Oncol.

[41] Paugh BS, Qu C, Jones C (2010). Integrated molecular genetic profiling of pediatric high-grade gliomas reveals key differences with the adult disease. J Clin Oncol Off J Am Soc Clin Oncol.

[42] Pollack IF, Stewart CF, Kocak M (2011). A phase II study of gefitinib and irradiation in children with newly diagnosed brainstem gliomas: a report from the Pediatric Brain Tumor Consortium. Neuro-Oncol.

[43] Poussaint TY, Kocak M, Vajapeyam S (2011). MRI as a central component of clinical trials analysis in brainstem glioma: a report from the Pediatric Brain Tumor Consortium (PBTC). Neuro-Oncol.

[44] Provenzani A, Fronza R, Loreni F (2006). Global alterations in mRNA polysomal recruitment in a cell model of colorectal cancer progression to metastasis. Carcinogenesis.

[45] Puget S, Blauwblomme T, Grill J (2012). Is biopsy safe in children with newly diagnosed diffuse intrinsic pontine glioma?. Am Soc Clin Oncol Educ Book ASCO Am Soc Clin Oncol Meet.

[46] Puget S, Philippe C, Bax DA (2012). Mesenchymal transition and PDGFRA amplification/mutation are key distinct oncogenic events in pediatric diffuse intrinsic pontine gliomas. PLoS One.

[47] Rickman DS, Millon R, De Reynies A (2008). Prediction of future metastasis and molecular characterization of head and neck squamous-cell carcinoma based on transcriptome and genome analysis by microarrays. Oncogene.

[48] Riemenschneider MJ, Koy TH, Reifenberger G (2004). Expression of oligodendrocyte lineage genes in oligodendroglial and astrocytic gliomas. Acta Neuropathol (Berl).

[49] Schorderet P, Lonfat N, Darbellay F (2013). A genetic approach to the recruitment of PRC2 at the HoxD locus. PLoS Genet.

[50] Schwartzentruber J, Korshunov A, Liu X-Y (2012). Driver mutations in histone H3.3 and chromatin remodelling genes in paediatric glioblastoma. Nature.

[51] Smyth GK (2004). Linear models and empirical Bayes methods for assessing differential expression in microarray experiments. Stat Appl Genet Mol Biol.

[52] Stock C, Schwab A (2014). Ion channels and transporters in metastasis. Biochim Biophys Acta.

[53] Sturm D, Witt H, Hovestadt V (2012). Hotspot mutations in H3F3A and IDH1 define distinct epigenetic and biological subgroups of glioblastoma. Cancer Cell.

[54] Szenker E, Ray-Gallet D, Almouzni G (2011). The double face of the histone variant H3.3. Cell Res.

[55] Tavazoie SF, Alarcón C, Oskarsson T (2008). Endogenous human microRNAs that suppress breast cancer metastasis. Nature.

[56] Taylor KR, Mackay A, Truffaux N (2014). Recurrent activating ACVR1 mutations in diffuse intrinsic pontine glioma. Nat Genet.

[57] Verhaak RGW, Hoadley KA, Purdom E (2010). Integrated genomic analysis identifies clinically relevant subtypes of glioblastoma characterized by abnormalities in PDGFRA, IDH1, EGFR, and NF1. Cancer Cell.

[58] Warren KE, Killian K, Suuriniemi M (2012). Genomic aberrations in pediatric diffuse intrinsic pontine gliomas. Neurooncol.

[59] Wu G, Broniscer A, McEachron TA (2012). Somatic histone H3 alterations in pediatric diffuse intrinsic pontine gliomas and non-brainstem glioblastomas. Nat Genet.

[60] Wu G, Diaz AK, Paugh BS (2014). The genomic landscape of diffuse intrinsic pontine glioma and pediatric non-brainstem high-grade glioma. Nat Genet.

[61] Zinn PO, Mahajan B, Majadan B (2011). Radiogenomic mapping of edema/cellular invasion MRI-phenotypes in glioblastoma multiforme. PLoS One.

