# Supplementary figures and images for: Histone H3F3A and HIST1H3B K27M mutations define two subgroups of diffuse intrinsic pontine gliomas with different prognosis and phenotypes
Source: Acta Neuropathol. 2015 Sep 23;130(6):815–27. doi: 10.1007/s00401-015-1478-0 (PMC4654747; doi:10.1007/s00401-015-1478-0)

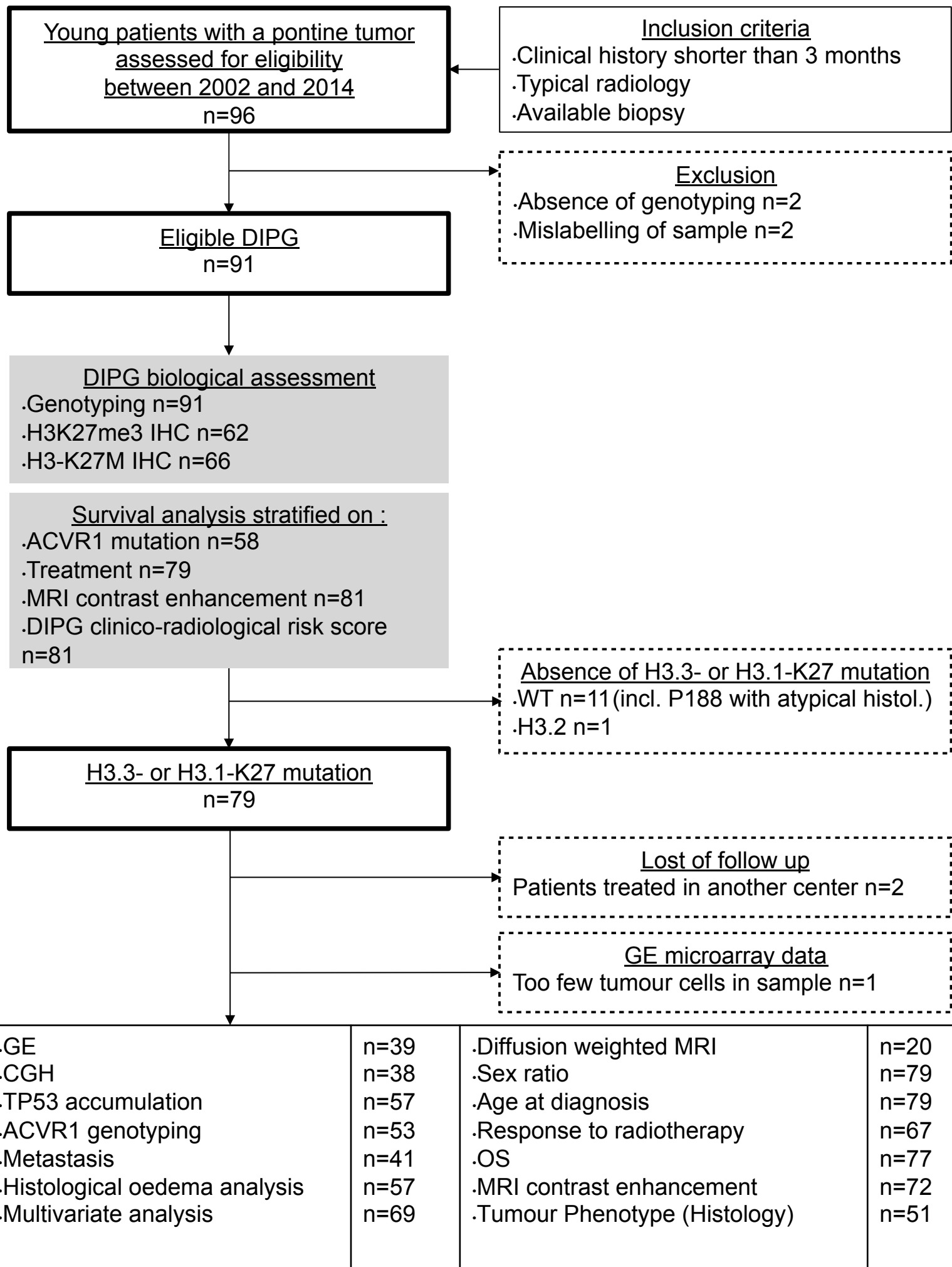

Supplement: Supplementary file 1 — Supplementary material 1: Figure S1 Flow diagram of patient selection process. Flow diagram describing the patient selection process and exclusion reasons for each statistical analysis. In bold frames, the patient counts, the grey backgrounds denote the statistical analysis and the dashed frames the exclusion criteria. (PDF 213 kb) [file 401_2015_1478_MOESM1_ESM.pdf]

a

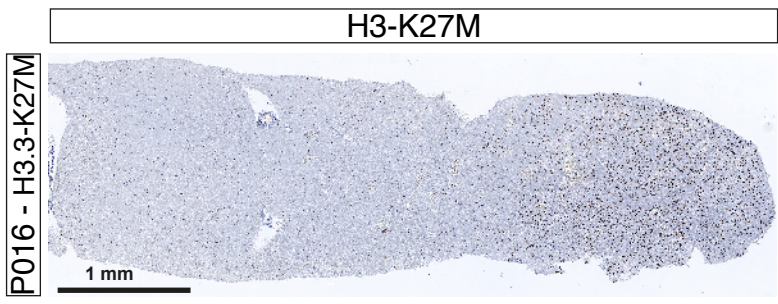

b

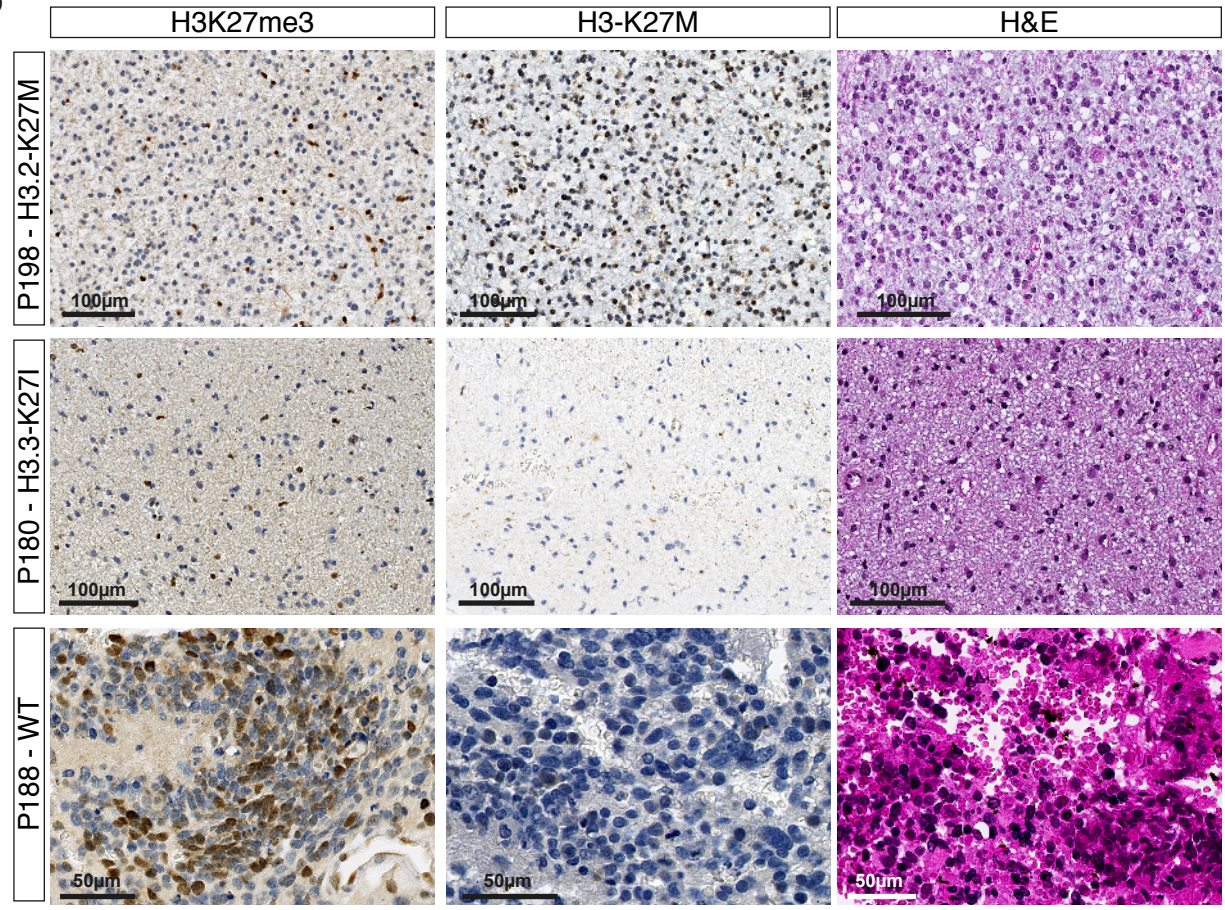

c

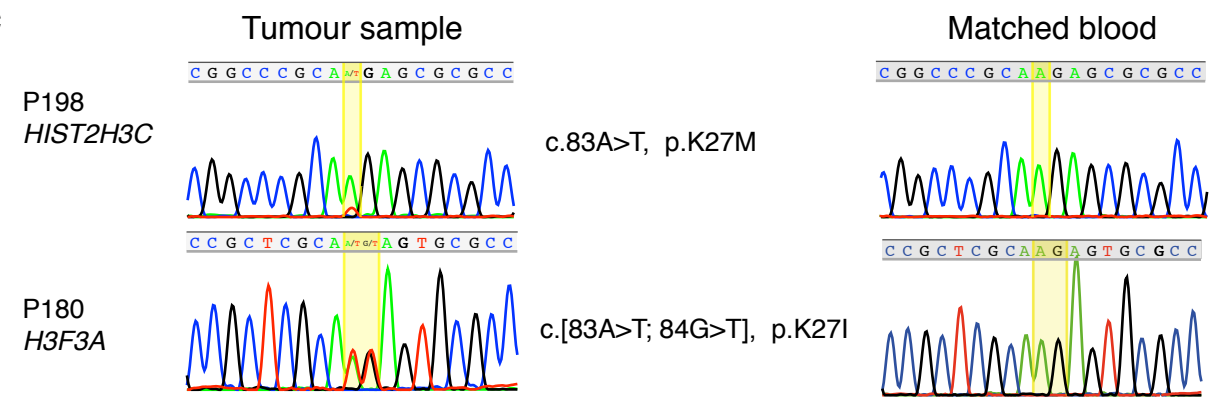

d

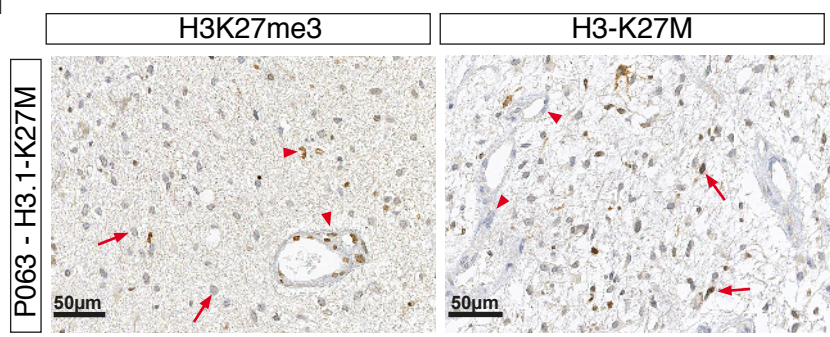

Supplement: Supplementary file 2 — Supplementary material 2: Figure S2 General and specific features of mutant H3K27 and H3K27me3 immunohistochemistry in DIPG. (a) H3-K27M IHC on an entire biopsy core from a H3.3 mutated DIPG (Scale bar: 1 mm). An important heterogeneity in H3-K27M detection can be observed along the same biopsy as shown by the important density of nuclei stained on the right side of the sample –reflecting an important infiltration of cancer cells, as opposed to the left side where isolated tumour cells can still be identified with the antibody against the mutant H3.3 protein. (b) Immunohistochemistry for H3K27me3 and H3-K27M as well as H&E staining are shown for the 3 atypical samples: P198, H3.2-K27M (Scale bar: 100 µm), P180, H3.3-K27I (Scale bar: 100 µm) and P188, WT DIPG samples (Scale bar: 50 µm). Both H3.2-K27M and H3.3-K27I samples are associated with a global loss of the trimethylation mark in a similar way to H3.1- and H3.3-K27M tumours. The H3.2 specimen is strongly stained by the antibody specific of K27M proteins as H3.3 tumours, unlike H3.3-K27I for which no staining was observed. In the P188 case, neither a trimethylation loss, nor a K27M staining was observed.. The histology of this sample exhibit features of a poorly differentiated GBM with pseudopalisading necrosis, mitosis and a giant cell component as seen on the H&E staining. (c) H3.2-K27M and H3.3-K27I somatic mutations. Sanger sequencing chromatograms showing the H3F3A double mutation encoding a p.K27I substitution and the HIST2H3C mutation encoding a p.K27M substitution in the indicated DIPG cases. Mutation positions are shown compared to matched normal DNA and indicated in yellow. (d) Representative results of IHC for H3K27me3 and H3-K27M for a H3.1-K27M mutated tumour biopsy. (Left panel) A strong reduction of overall H3K27me3 is observed with a specific loss of the trimethylation mark in tumour glial cells as revealed by nuclei blue counterstaining (arrows) where the nuclei of normal cells remain labelled i [file 401_2015_1478_MOESM2_ESM.pdf]

a

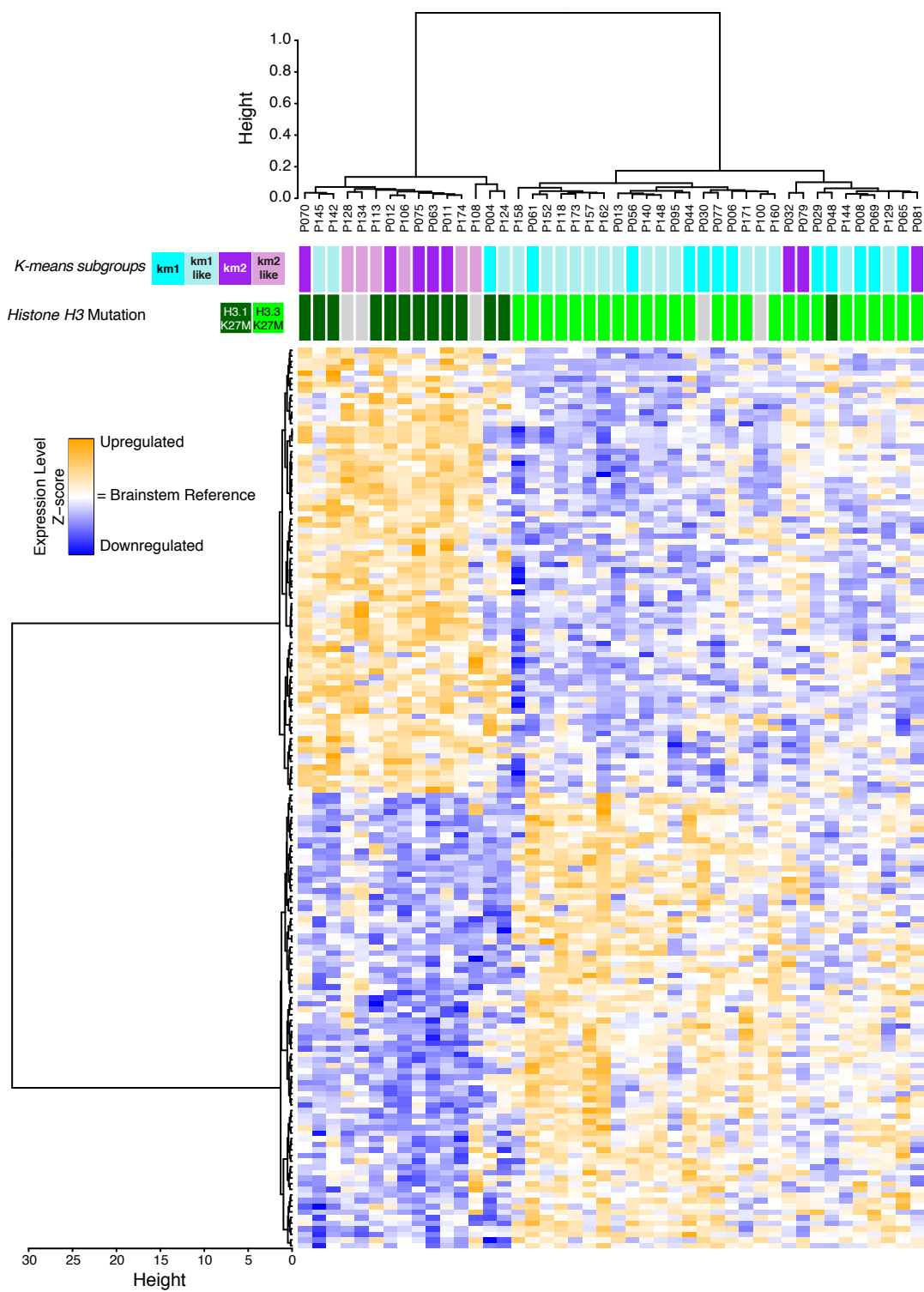

Supplement: Supplementary file 3 — Supplementary material 3. Figure S3A Molecular signature of H3.1 and H3.1 subgroups. (a) Unsupervised hierarchical clustering and heatmap associated with the gene expression profile of the 160 differentially expressed genes (Benjamini & Hochberg adjusted p-value < 0.05) between H3.1 and H3.3 subgroups. The mutational status of histone H3F3A (light green), HIST1H3B (dark green) or WT for those two genes as well as HIST2H3C (grey) are reported above the heatmap. The affiliation (plain colour) or similarity (light colour) of each sample to the two K-means subgroups identified in a previous study are indicated in blue for km1 and purple for km2 [46]. Overall, H3.1 mutated samples are preferentially associated with a km2 signature (8/13 DIPG samples) whereas H3.3 preferentially show a km1 expression profile (23/26 samples, fisher’s exact test, p-value = 0.0021). (PDF 229 kb) [file 401_2015_1478_MOESM3_ESM.pdf]

b

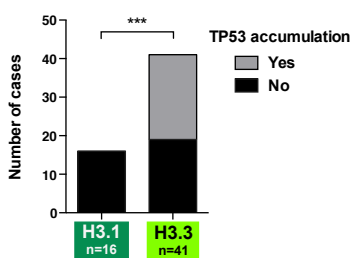

c

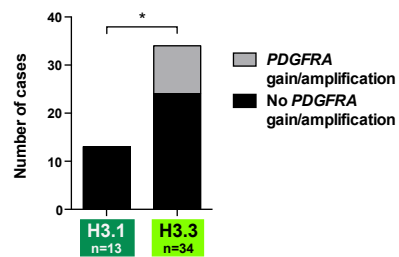

d

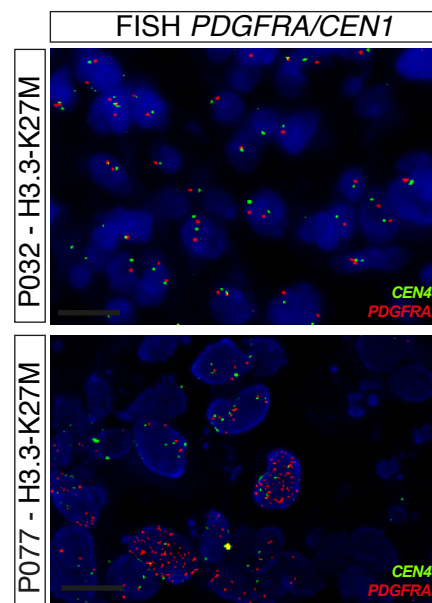

e

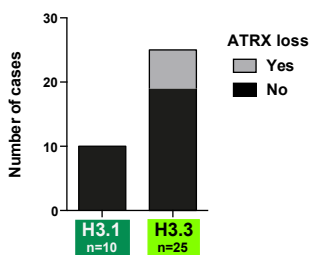

f

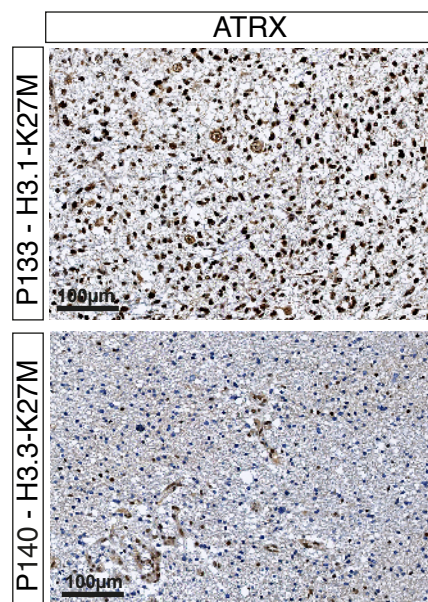

g

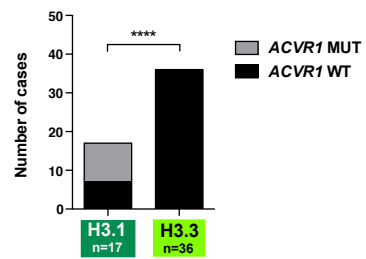

Supplement: Supplementary file 4 — Supplementary material 4: Figure S3B Molecular signature of H3.1 and H3.1 subgroups. (b) TP53 protein was assessed by IHC on 57 DIPG biopsies. p53 accumulation was restricted to the H3.3 mutated subgroup (p-value = 0.0001; fisher exact test, top panel). A gene signature comprising upregulated targets of TP53 in a cancer cell line strongly enriched among H3.1 tumours [30], pointing to an inactivation of TP53 transcriptional activity in H3.3-mutated samples (bottom panel). (c) Genomic alterations (amplification or gain) of PDGFRA identified by aCGH & FISH analyses were found exclusively in H3.3-K27M samples (p-value = 0.428; two-sided fisher exact test, top panel). Genes preferentially upregulated in ‘PDGFRA-amplified pHGG’ [41] were significantly overexpressed in H3.3 vs. H3.1 tumours as shown on the GSEA enrichment plot (bottom panel). (d) Representative results of PDGFRA/CEN4 FISH showing no amplification (top panel) or often innumerable red PDGFRA signals, but only a few green CEN4 signals per nucleus indicating a PDGFRA gene amplification (bottom panel; 600X magnification). (e) Bar graphs showing an exclusive loss of ATRX expression in H3.3-K27M tumours (p-value = 0.152, two-sided fisher exact test). (f) Representative results of IHC for ATRX in H3.1/3-K27M mutated tumour biopsies. (Top panel) A strong nuclear staining is observed in all tumour and normal cells. (Bottom panel) A loss of ATRX staining is observed in tumour glial cell nuclei as revealed by nuclei blue counterstaining while the nuclei of normal cells remain labelled in brown (for example endothelial cells). (Scale bar: 100 µm). (g) Bar graphs showing segregation of activating mutations in ACVR1 with H3.1-K27M mutation (p-value < 0.0001, two-sided fisher exact test) in a series of 53 DIPG cases. (PDF 850 kb) [file 401_2015_1478_MOESM4_ESM.pdf]

a

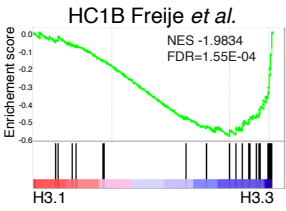

b

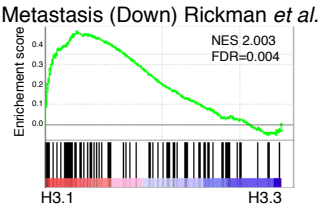

c

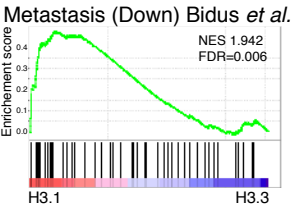

d

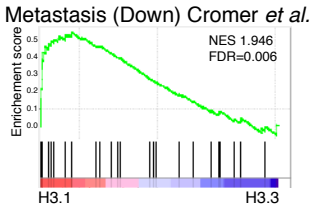

e

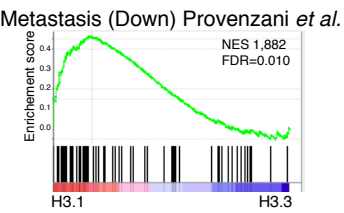

f

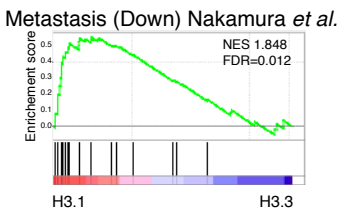

g

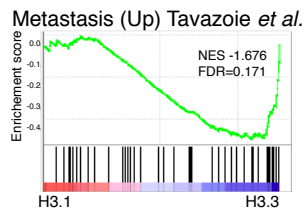

Supplement: Supplementary file 5 — Supplementary material 5: Figure S4A Recurrent molecular alterations in H3.3-K27M DIPG and metastases identified by imaging. (a) GSEA analysis demonstrated that DIPG with H3.3-K27M mutation are significantly enriched in the ‘neuronal’ gene set reported in Freije et al. (HC1B gene set) [21]. (b-g) GSEA analysis showed that the top genes inhibited in metastatic vs. non-metastatic head and neck squamous cell carcinoma tumour [47] (b-c), endometrial tumours [4] (d), colorectal carcinoma cells [44] (e), pancreatic cancer cells [38] (f) were also downregulated in H3.3 vs. H3.1 subgroups. Conversely, top genes induced in metastatic breast adenocarcinoma [55] were upregulated in H3.3 vs. H3.1 tumours (g). (PDF 176 kb) [file 401_2015_1478_MOESM5_ESM.pdf]

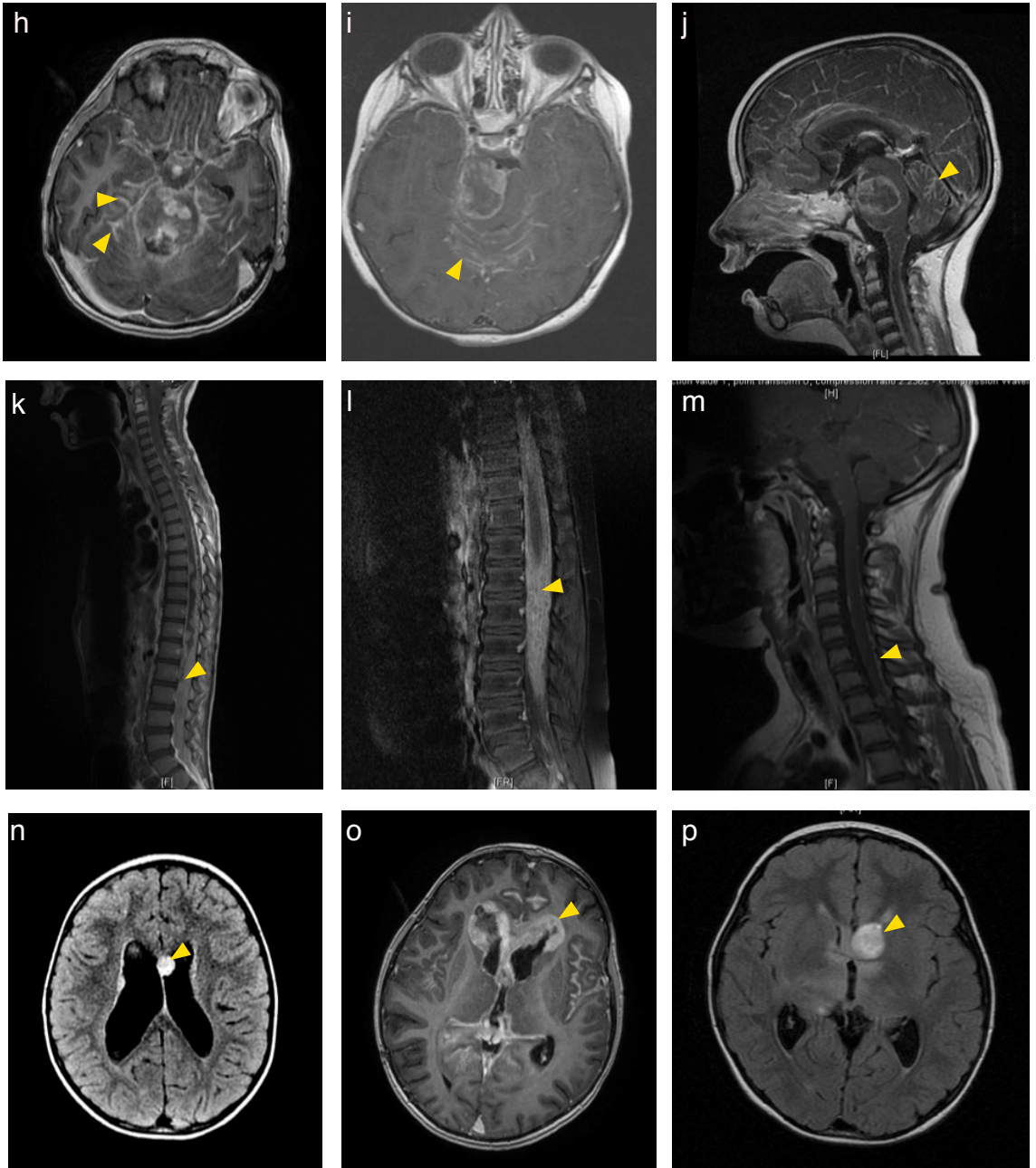

q

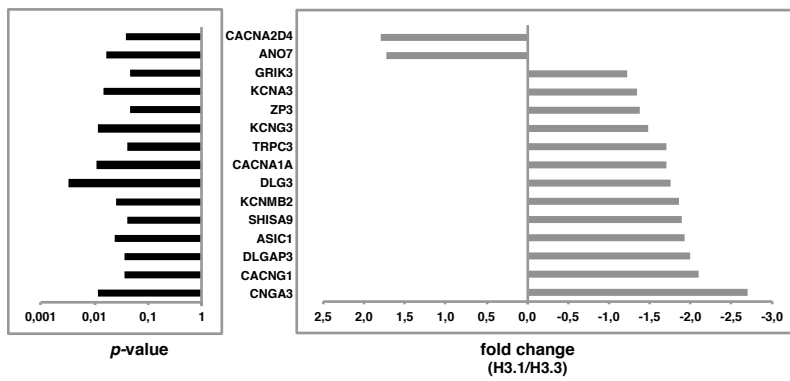

Supplement: Supplementary file 6 — Supplementary material 6: Figure S4B Recurrent molecular alterations in H3.3-K27M DIPG and metastases identified by imaging. (h-p) Metastases in H3.3 mutated DIPG were assessed on MRI performed within the last two months prior to death. Two distinct patterns were observed. Firstly, a linear contrast enhancement of the meninges was apparent, indicating either a loco-regional leptomeningeal spread in the cerebellum or in the cerebrum (h, i, j) or a distant leptomeningeal spread in the spinal cord (k, l, m). Second, a subependymal spread forming nodules in the ventricles was visible as hypersignal on diffusion-weighted imaging (n), T1-weighted sequences with gadolinium (o) or FLAIR imaging (p). (q) Gene list functional enrichment using TOPPFun (https://toppgene.cchmc.org/) of the list of 160 differentially expressed genes between the two tumour subgroups highlighted ‘gated channel activity’ (GO:0022836, p-value = 1.06 e-6 and Benjamini & Hochberg FDR = 2.893 e-4) and ‘ion channel activity’ (GO:0005216, p-value = 1.101 e-5 and Benjamini & Hochberg FDR = 1.567 e-3) molecular functions as well as ‘cation channel complex’ cellular component (GO:0005261, p-value = 3.413 e-6 and Benjamini & Hochberg FDR = 4.659 e-4) as significantly enriched. The majority of the genes involved in those categories are downregulated in H3.1 vs. H3.3 samples as reflected by their fold change and corresponding p-value. (PDF 716 kb) [file 401_2015_1478_MOESM6_ESM.pdf]

a

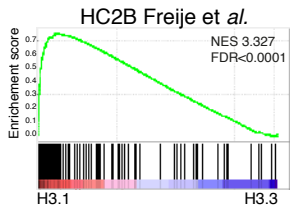

b

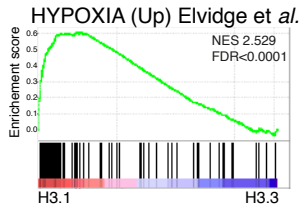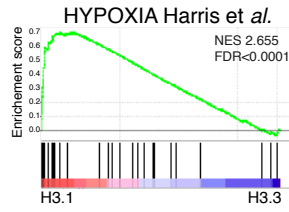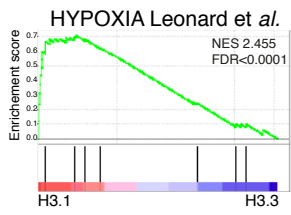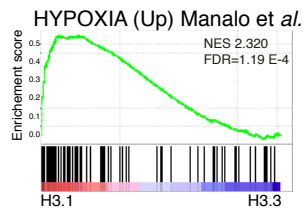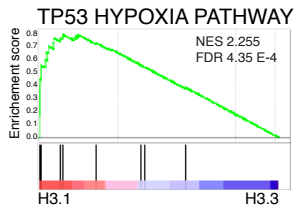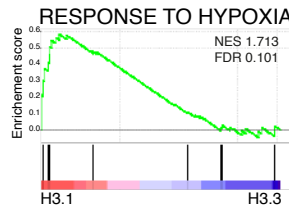

c

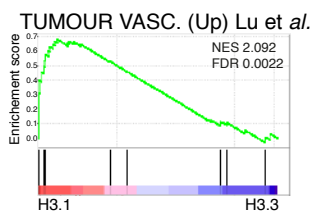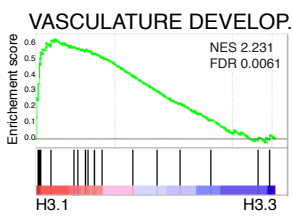

Supplement: Supplementary file 7 — Supplementary material 7: Figure S5 Recurrent molecular alterations in H3.1-K27M DIPG. (a) GSEA analysis demonstrated that upregulated genes in DIPG with H3.1-K27M mutation are significantly enriched in the ‘mesenchymal’ gene set reported in Freije et al. (HC2B gene set) [21]. (b) Hypoxic characteristic of H3.1 tumours was revealed by a significant enrichment of several hypoxia-related gene sets reinforcing the results presented in Fig. 3b, representing upregulated genes under hypoxic conditions or after HIF1A overexpression [18, 27, 30, 33, 36] as well as the ‘response to hypoxia’ biological process (GO:0001666) and ‘Biocarta P53 hypoxia’ pathway. (c) In addition to the angiogenesis pathway both ‘vasculature development’ biological process (GO:0001944) and genes identified in processes related to ‘tumour vascularization’ [35] were found enriched in upregulated DEGs in H3.1-mutated samples. The normalized enrichment score (NES) and false discovery rate (FDR)-corrected q-value are indicated. (PDF 196 kb) [file 401_2015_1478_MOESM7_ESM.pdf]

a

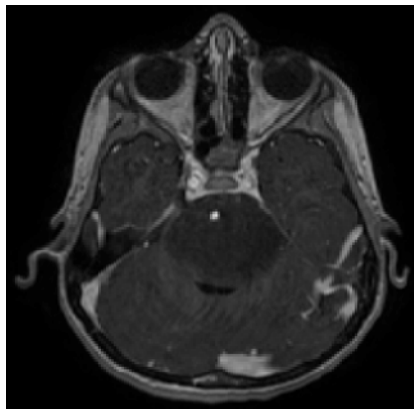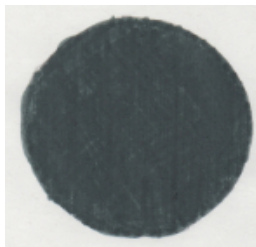

b

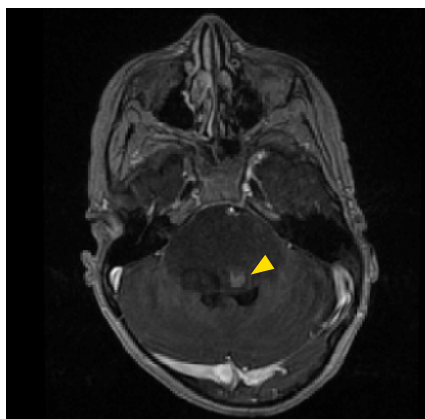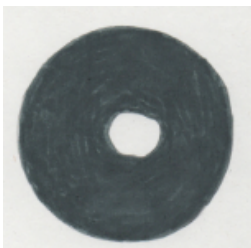

c

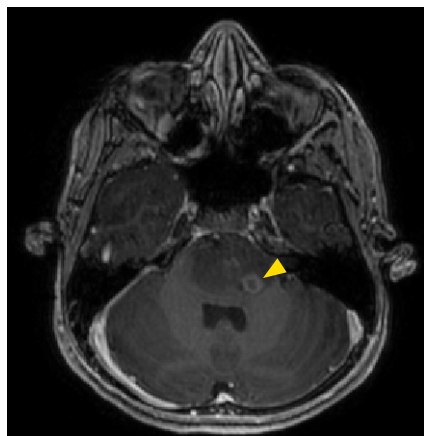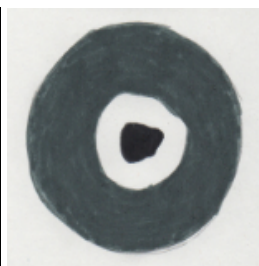

d

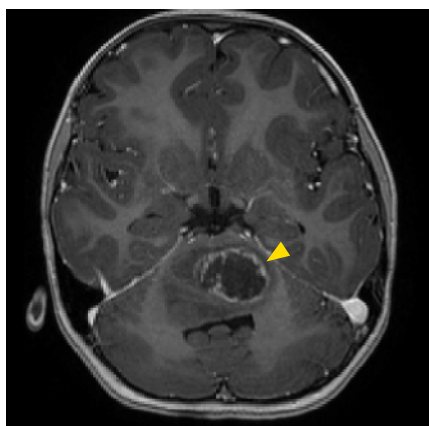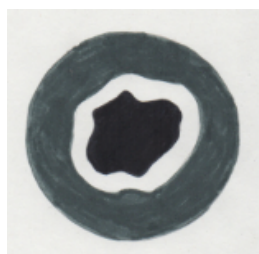

Supplement: Supplementary file 8 — Supplementary material 8: Figure S6 Radiological classification of neoangiogenesis and necrosis. Contrast enhancement indicative of neoangiogenesis and necrosis were graded on T1 + contrast MRI sequences acquired on the axial plane for each patient and indicated by an arrow on the left side and schematized on the right. Images are displayed that represent each configuration in terms of (a) absence of contrast, (b) nodular contrast enhancement, (c) ring contrast enhancement, (d) large necrotic area with ring enhancement. (PDF 449 kb) [file 401_2015_1478_MOESM8_ESM.pdf]

# Supplemental Figure S7 Castel et al.

a

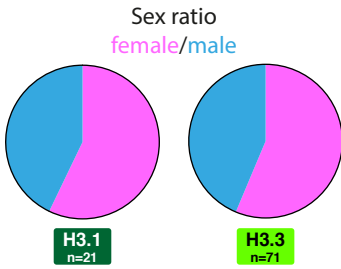

b

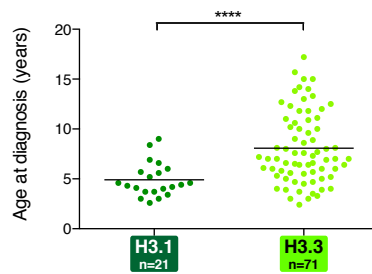

c

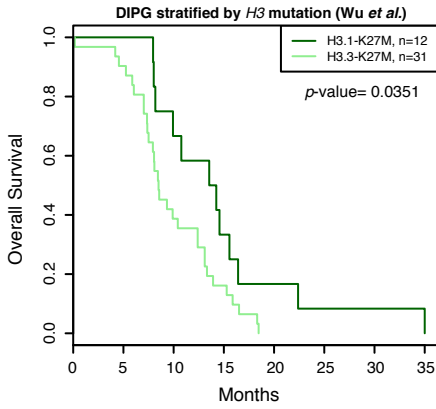

d

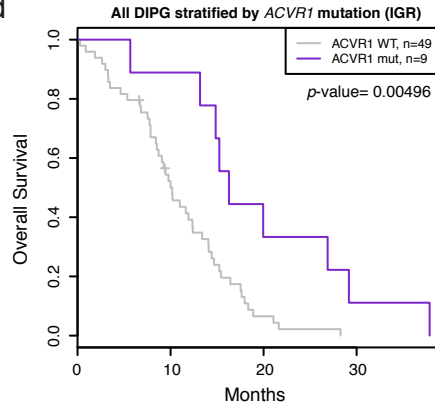

e

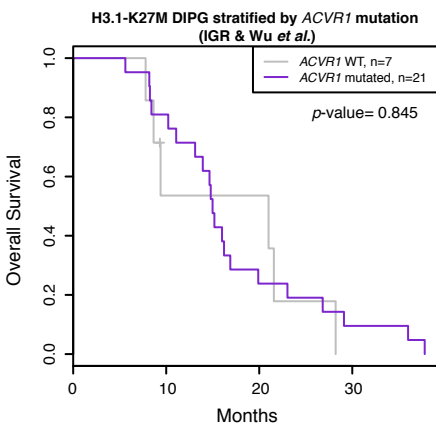

f

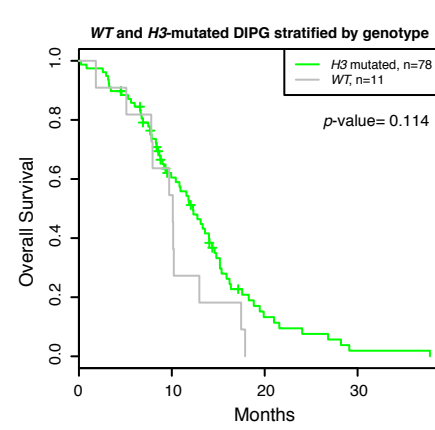

g

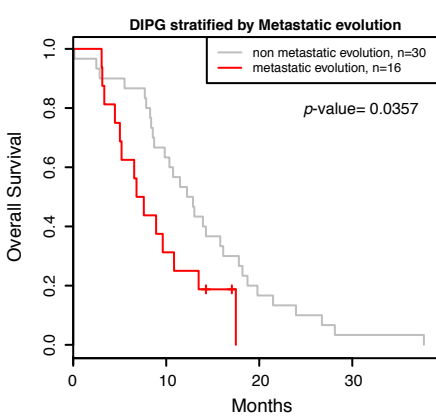

h

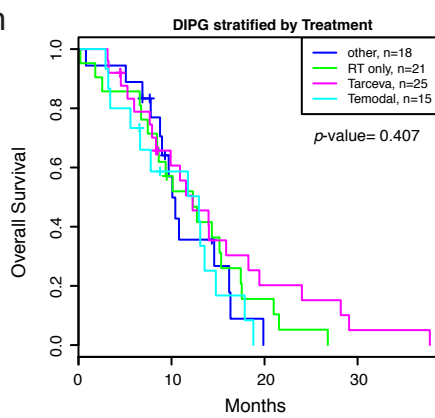

i

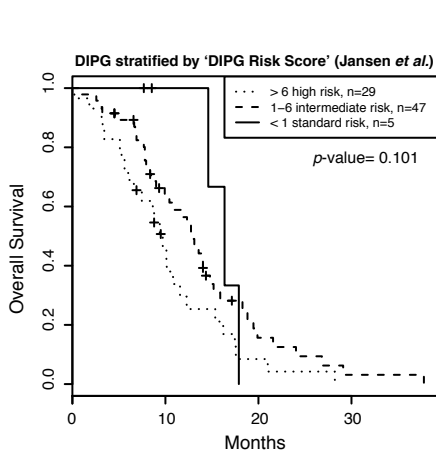

j

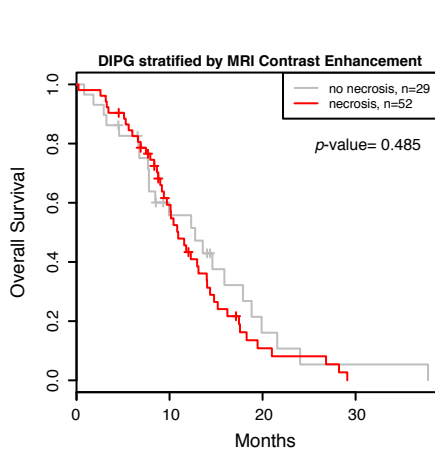

Supplement: Supplementary file 9 — Supplementary material 9: Figure S7 In-depth analysis of clinical parameters differentiating DIPG subgroups. (a) Sex distribution of cases with H3.1 and H3.3 mutations in a cohort of 92 DIPG from published datasets [8, 20, 60]. No difference of sex proportion was observed. (b) Age distribution of H3.1 and H3.3 patients at diagnosis. The median age at diagnosis was significantly smaller in H3.1- (4.4 years, n = 21) in comparison with H3.3-mutated samples (7.0 years, n = 71) from published studies [8, 20, 60] as observed in our cohort (Fig. 5b). Statistical significance was calculated with Mann–Whitney test (median, ****p-value < 0.0001). (c) Kaplan–Meier estimates of the survival stratified by H3.1 or H3.3-K27M mutations in the published data from Wu et al. [60]. H3.1 tumours (n = 12) are associated with a better overall survival than H3.3 tumours (n = 31) (median survival times of 14.29 and 8.74 months, respectively; p-value = 0.0351; log-rank test). (d-e) Overall survival curves of all ACVR1 mutated or ACVR1 WT tumours in our cohort highlight an increased median survival time for mutated samples (d, median survival times of 16.21 and 9.71 months respectively; p-value = 0.00496; log-rank test). However, no difference was observed among the H3.1 tumour subgroup between ACVR1 WT and ACVR1 mutated samples (e, p-value = 0.845; log-rank test, data compiled from our cohort with a previously published one [60] ). (f) Overall survival curves of all DIPG from the cohort stratified by H3-K27M mutation or WT status. No difference in outcome was associated with H3-K27M tumours (n = 78) vs. the WT tumours (n = 11) (p-value = 0.114; log-rank test). (g) Influence of a metastatic evolution on DIPG overall survival curves. A shorter overall survival was observed for patients presenting metastasis development during the course of the disease (n = 16) compared to the others (n = 30, p-value = 0.0357; log-rank test). (h) Overall survival curves of patients with DIPG stratified accordin [file 401_2015_1478_MOESM9_ESM.pdf]
