# Supplementary material for: Histone H3F3A and HIST1H3B K27M mutations define two subgroups of diffuse intrinsic pontine gliomas with different prognosis and phenotypes
Source: Acta Neuropathol. 2015 Sep 23;130(6):815–27. doi: 10.1007/s00401-015-1478-0 (PMC4654747; doi:10.1007/s00401-015-1478-0)
Supplement: Supplementary file 10 — Supplementary material 10: Table S1: Patient summary data. Clinical, histopathological, radiological and molecular variables in the cohort of 91 DIPG of the study. “FU”: Follow-up in month; “DIPG_survival model”: score according to the model in Jansen et al.; “DIPG survival model 3 classes”: classification into 3 classes of low intermediate and high risk according to Jansen et al.; “Metastasis”: presence or absence of metastases identified by MRI within the last 3 months of life; “Km1/Km2 Group” : belonging to the Km1 or Km2 groups based on gene expression profile as defined in Puget et al; “Tumour Compartment Oligo/Astro” : Oligodendroglial or astrocytic differentiation of the tumour cells; “WHO” : grading according to the WHO classification; “CHSA” : grading according to the Sainte-Anne Hospital classification; “Oedema”: presence of extracellular oedema on histological sections; “Type of Contrast Enhancement (MRI)”: classification in 4 classes of CE as defined in suppl. Fig S6; “NA”: Data not available; “NI”: Non-interpretable. Table S2: PCR primers. Table S3: List of differentially expressed genes. Genes significantly differentially expressed between H3.1 and H3.3 tumour subgroups as determined by moderated t-test using an adjusted p-value cutoff of 5 % are indicated with their corresponding ratio and p-value. Genes in bold correspond to those with an adjusted p-value cutoff of 1 %. (PDF 167 kb) [file 401_2015_1478_MOESM10_ESM.pdf]

Suppl. Table S1: Patient summary data.

ID patient	Gender	age_at diagnosis years	FU month	Status deceased=1	DIPG_survival model Jansen_et_al.	DIPG_survival model 3_classes	Response_to radiotherapy	Treatment Type	Metastasis	CGH_Array Name	GE_Array Name	chr1q GAIN	chr2 GAIN	chr4q12 GAIN_or AMPLI	chr14q24 LOSS	chr17p13 LOSS	Km1/Km2 group	*H3* mutation	H3-K27M IHC	H3K27me3 IHC	*ACVR1* mutation	*ACVR1* mutation status	TP53 accumulation IHC	ATRX Expression IHC	Tumour Compartment Oligo/Astro	MIB-1 PROLIF. INDEX %	GAIN/AMPLI. *PDGFRA* FISH	PDGFRa GAIN FISH+aCGH	WHO	CHSA	Oedema (Histology)	Constrast Enhancement (MRI)	Type_of_Constrast Enhancement (MRI)
P004	F	7.93	26.82	1	6	intermediate	good	Radiotherapy	0	28068	11955_1_1	yes	no	no	yes	no	KM1	K27M H3.1	1	NA	G328W	1	0	NA	NA	NA	NA	0	DIPG	DIPG	Low	1	Nodular CE
P006	M	6.58	3.43	1	7	high	good	Temodal	1	14175_3	27014_4	yes	no	no	no	no	KM1	K27M H3.3	1	0	wild-type	0	0	NA	astro	50	NA	0	astro IV	TGNM	Low	1	Large necrotic area
P008	M	5.07	3.21	1	3	intermediate	good	Temodal	1	14175_4	27014_3	yes	no	yes	yes	yes	KM1	K27M H3.3	1	0	wild-type	0	1	0	oligo	30	NI	1	astro II	oligoastro A	Low	0	None
P011	F	5.08	13.11	1	3	intermediate	good	Temodal	0	14173_1	19731_2_3	yes	no	no	no	no	KM2	K27M H3.1	1	0	G328E	1	0	1	astro	50	0	0	astro IV	TGNM	Medium	1	Large necrotic area
P012	F	3.63	14.79	1	6	intermediate	good	Temodal	NA	14173_2	11958_1_1	yes	yes	no	no	no	KM2	K27M H3.1	NA	NA	G328V	1	0	NA	astro	50	NA	0	astro IV	GBM	Medium	1	Large necrotic area
P013	F	7.46	6.64	1	2	intermediate	poor	Temodal	1	20808_3	19731_2_4	no	no	no	no	no	KM1_LIKE	K27M H3.3	1	0	wild-type	0	1	NA	oligo	40	NA	0	astro II	oligo A	Low	0	None
P016	M	9.3	11.79	1	5	intermediate	good	Temodal	NA	20755_3	NA	no	no	no	no	no	NA	K27M H3.3	1	0	NA	NA	0	1	oligo	30	0	0	oligo astro III	oligo astro B	Low	1	Ring CE
P025	M	4.48	12.32	1	1	intermediate	good	Radiotherapy	0	26673	NA	yes	yes	yes	NA	no	NA	K27M H3.3	1	1	wild-type	0	0	NA	NA	10	NA	1	DIPG	DIPG	Medium	0	None
P026	M	7.04	13.32	1	NA	NA	NA	NA	NA	26672	NA	no	no	no	no	no	NA	K27M H3.3	NA	NA	wild-type	0	1	NA	NA	NA	NA	0	DIPG	DIPG	Low	NA	NA
P028	F	6.21	6.61	1	11	high	NA	Radiotherapy	NA	20755_1	NA	yes	no	yes	no	yes	NA	K27M H3.3	NA	NA	NA	NA	NA	NA	NA	NA	NA	1	NA	NA	NA	1	Ring CE
P029	F	3.57	15.36	1	11	high	NA	Radiotherapy	NA	NA	11960_1_3	NA	NA	NA	NA	NA	KM1	K27M H3.3	0	0	wild-type	0	0	NA	oligo	30	NA	NA	oligo astro III	oligo B	High	NA	NA
P030	M	12.98	10.11	1	7	high	good	Other	NA	26444	12016_1_1	no	no	yes	no	no	KM1	wild-type	1	NA	wild-type	0	NA	NA	NA	30	NA	1	oligo astro III	oligo astro B	Low	1	Large necrotic area
P032	M	12	18.29	1	6	intermediate	good	Tarceva	0	15797_2	27014_1	NA	NA	NA	NA	NA	KM2	K27M H3.3	1	0	wild-type	0	1	1	oligo	50	0	0	oligo astro III	oligo astro B	High	1	Large necrotic area
P043	M	9.2	1.82	1	7	high	poor	Radiotherapy	NA	15797_3	NA	no	no	no	yes	yes	NA	wild-type	NA	NA	wild-type	0	NA	NA	NA	NA	NA	0	NA	NA	NA	0	None
P044	F	9.57	12.29	1	7	high	good	Tarceva	NA	14170_1	19737_1_4	yes	no	no	no	no	KM1	K27M H3.3	1	0	wild-type	0	1	0	oligo	40	NI	0	oligo astro III	oligo astro B	Low	1	Ring CE
P048	F	6.29	7.79	1	3	intermediate	good	Temodal	0	14170_2	19719_1_2	no	no	no	no	no	KM1	K27M H3.1	1	0	wild-type	0	0	NA	astro	30	NA	0	astro III	oligo astro A	Medium	0	None
P050	M	6.43	6.75	1	6	intermediate	good	Radiotherapy	NA	25096	NA	no	no	no	yes	yes	NA	K27M H3.3	NA	NA	wild-type	0	NA	NA	NA		NA	0	NA	NA	NA	0	None
P052	M	4.76	7.79	1	3	intermediate	NA	Other	NA	15797_4	NA	no	no	no	no	yes	NA	wild-type	1	NA	wild-type	0	1	NA	NA	20	NA	0	DIPG	DIPG	Medium	0	None
P056	M	10.87	18.82	1	3	intermediate	poor	Temodal	0	15798_1	19720_2_2	no	no	no	yes	no	KM1	K27M H3.3	1	0	wild-type	0	1	0	oligo	30	NI	0	oligo astro III	oligo astro B	Low	0	None
P059	M	10.75	NA	1	3	intermediate	NA	Temodal	NA	20755_4	NA	yes	yes	no	no	yes	NA	K27M H3.1	1	0	G328E	1	0	NA	oligo	30	NA	0	astro II	oligo A	High	0	None
P061	F	11.74	2.57	1	5	intermediate	poor	Radiotherapy	0	14173_4	19747_2_1	no	no	no	yes	no	KM1	K27M H3.3	1	0	wild-type	0	0	1	oligo	30	1	1*	oligo astro III	oligo astro B	High	1	Nodular CE
P062	M	11.66	6.57	0	3	intermediate	poor	Temodal	NA	15798_2	NA	no	no	no	yes	no	NA	K27M H3.3	1	0	wild-type	0	0	NA	NA	30	NA	0	DIPG	DIPG	Low	0	None
P063	F	5.98	5.61	1	7	high	good	Temodal	0	NA	19747_2_3	NA	NA	NA	NA	NA	KM2	K27M H3.1	1	0	G328E	1	0	1	astro	30	NA	NA	astro IV	TGNM	Medium	1	Large necrotic area
P065	M	10.57	2.93	1	3	intermediate	good	Temodal	0	15798_3	19748_2_3	no	no	no	no	yes	KM1	K27M H3.3	1	0	wild-type	0	1	0	oligo	30	NI	0	astro III	oligo astro B	Medium	0	None
P069	F	6.76	5.29	1	7	high	good	Tarceva	1	14174_2	27049_1	yes	yes	no	yes	yes	KM1	K27M H3.3	1	0	wild-type	0	1	1	astro	50	NI	0	astro IV	TGNM	Low	1	Large necrotic area
P070	M	5.02	8.64	1	11	high	good	Radiotherapy	0	26578	12025_1_1	yes	yes	no	NA	no	KM2	K27M H3.1	1	0	wild-type	0	0	1	astro	30	NI	0	astro III	NA	Medium	1	Nodular CE
P072	F	13.64	7.46	1	11	high	poor	Radiotherapy	NA	14174_3	19768_2_3	no	no	no	yes	no	KM2	K27M H3.3	1	0	wild-type	0	1	1	astro	20	0	0	astro IV	TGNM	Low	1	Large necrotic area
P075	F	4.4	21	1	11	high	good	Radiotherapy	NA	NA	12290_1_3	NA	NA	NA	NA	NA	KM2	K27M H3.1	1	0	wild-type	0	0	1	astro	50	NI	NA	astro IV	TGNM	Medium	1	Large necrotic area
P077	M	7.63	0.21	1	7	high	poor	Radiotherapy	0	14175_1	27048_1	yes	no	yes	yes	no	KM1	K27M H3.3	1	0	wild-type	0	1	1	oligo	50	0	1*	astro IV	oligo astro B	Low	1	Nodular CE
P079	M	7.45	15.18	1	NA	NA	NA	NA	NA	NA	12291_1_2	NA	NA	NA	NA	NA	KM2	K27M H3.3	1	1	wild-type	0	1	1	astro	20	NI	NA	astro IV	GBM	Low	NA	NA
P081	M	6.69	17.57	1	11	high	good	Radiotherapy	1	15798_4	19769_2_2	no	no	no	yes	yes	KM2	K27M H3.3	1	NA	wild-type	0	1	NA	astro	50	NA	0	astro IV	GBM	Low	1	Nodular CE
P083	F	11.21	14.36	1	6	intermediate	poor	Radiotherapy	0	14175_2	NA	no	no	no	no	no	NA	K27M H3.3	1	0	wild-type	0	1	NA	oligo	10	NA	0	astro II	oligo B	Medium	1	Nodular CE
P095	M	5.9	10.93	1	4	intermediate	poor	Tarceva	1	NA	19731_1_3	NA	NA	NA	NA	NA	KM1_LIKE	K27M H3.3	1	0	wild-type	0	1	1	oligo	NA	NI	NA	astro II	NA	Medium	1	Ring CE
P096	F	3.89	14.04	1	6	intermediate	good	Tarceva	0	NA	NA	NA	NA	NA	NA	NA	NA	K27M H3.3	1	0	wild-type	0	0	1	astro	40	0	0	astro IV	TGNM	Low	1	Large necrotic area
P097	M	10.33	3.25	1	7	high	poor	Tarceva	NA	NA	NA	NA	NA	NA	NA	NA	NA	K27M H3.3	1	0	wild-type	0	1	NA	oligo	40	NA	NA	oligo astro III	NA	Low	1	Large necrotic area
P100	F	4.71	12.96	1	3	intermediate	good	Temodal	0	NA	19731_2_2	NA	NA	NA	NA	NA	NA	wild-type	1	0	NA	NA	1	1	NA	20	0	0	astro II	NA	Low	1	Ring CE
P106	M	3.3	29.11	1	3	intermediate	good	Tarceva	NA	28843_3	19732_2_3	yes	yes	no	no	no	KM2_LIKE	K27M H3.1	1	0	G328E	1	0	1	oligo	40	NI	0	oligo astro III	oligo astro B	Medium	1	Large necrotic area
P108	F	16.29	17.89	1	-1	standard	poor	Temodal	0	28835_3	19732_2_4	no	no	yes	yes	yes	KM2_LIKE	wild-type	0	0	wild-type	0	0	1	NA	10	1	1	astro III	oligo astro B	Low	0	None
P112	F	12.74	10.21	1	NA	NA	NA	NA	NA	NA	NA	NA	NA	NA	NA	NA	NA	wild-type	1	0	NA	NA	1	NA	NA	50	NA	NA	astro IV	TGNM	Low	NA	NA
P113	F	5.87	28.21	1	7	high	good	Tarceva	0	28842_4	19733_2_1	no	yes	no	yes	no	KM2_LIKE	K27M H3.1	1	0	wild-type	0	0	NA	astro	NA	NA	0	astro IV	TGNM	Medium	1	Large necrotic area
P116	M	4.38	9.71	1	7	high	good	Other	1	NA	NA	NA	NA	NA	NA	NA	NA	wild-type	0	0	wild-type	0	1	NA	NA	NA	NA	NA	astro II	NA/pilo	NA	1	Large necrotic area
P118	M	5.75	4.57	1	3	intermediate	good	Tarceva	1	NA	19737_1_1	NA	NA	NA	NA	NA	KM1_LIKE	K27M H3.3	1	0	wild-type	0	0	1	oligo	40	1	1	astro III	oligo A	High	0	None
P120	M	14.71	15.89	1	2	intermediate	poor	Tarceva	0	NA	NA	NA	NA	NA	NA	NA	NA	K27M H3.3	1	0	NA	NA	0	NA	NA	NA	NA	NA	DIPG	DIPG	NA	0	None
P122	M	9.3	24.04	1	3	intermediate	poor	Tarceva	0	NA	NA	NA	NA	NA	NA	NA	NA	K27M H3.3	1	0	NA	NA	1	NI	oligo	20	0	0	astro II	NA	Medium	0	None
P123	F	6.62	8.46	1	6	intermediate	poor	Radiotherapy	0	NA	NA	NA	NA	NA	NA	NA	NA	K27M H3.3	1	0	wild-type	0	1	1	oligo	70	0	0	oligo astro III	oligoastro B	Medium	0	None
P124	M	8.65	21.57	1	6	intermediate	good	Radiotherapy	0	28803_3	19737_2_2	yes	yes	no	no	no	KM1_LIKE	K27M H3.1	1	0	wild-type	0	0	1	astro	10	0	0	astro II	oligoastro A	Medium	0	None
P125	M	4.9	15.18	1	6	intermediate	good	Radiotherapy	NA	NA	NA	NA	NA	NA	NA	NA	NA	K27M H3.1	1	0	G356D	1	0	NA	oligo	30	NA	NA	astro III	oligo B	Low	1	Nodular CE
P128	M	12.12	10.14	1	10	high	good	Radiotherapy	NA	28841_2	19719_2_1	yes	no	no	no	no	KM2_LIKE	wild-type	0	0	wild-type	0	0	NA	NA	30	NA	0	astro IV	GBM	Medium	1	Large necrotic area
P129	F	7.78	7.68	1	3	intermediate	poor	Tarceva	1	NA	19719_2_2	NA	NA	NA	NA	NA	KM1_LIKE	K27M H3.3	1	0	wild-type	0	0	1	oligo	40	1	1	astro III	oligo astro A	Medium	0	None
P133	F	6.9	19.89	1	1	intermediate	good	Other	0	NA	NA	NA	NA	NA	NA	NA	NA	K27M H3.1	1	0	G328V	1	0	1	astro	10	0	0	astro II	oligo astro A	High	0	None
P134	M	11.7	17.46	1	11	high	NA	Radiotherapy	NA	28841_3	19720_1_1	yes	no	no	no	no	KM2_LIKE	wild-type	1	0	wild-type	0	0	NA	NA	60	0	0	astro IV	GBM	Medium	1	Ring CE
P139	M	8.77	9	1	2	intermediate	poor	Other	1	NA	NA	NA	NA	NA	NA	NA	NA	K27M H3.3	1	0	wild-type	0	0	NA	oligo	30	NA	NA	astro III	oligo astro B	Low	1	Ring CE
P140	M	9.39	8.36	1	6	intermediate	good	Tarceva	0	NA	19720_1_4	NA	NA	NA	NA	NA	KM1_LIKE	K27M H3.3	1	0	wild-type	0	1	0	oligo	20	1	1	oligoastro III	oligoastro B	Low	1	Ring CE
P141	M	13.99	10.82	1	7	high	poor	Other	0	NA	NA	NA	NA	NA	NA	NA	NA	K27M H3.3	NA	NA	NA	NA	NA	NA	NA	20	NA	NA	astro III	NA	Low	1	Ring CE
P142	F	5.74	16.21	1	7	high	good	Other	0	28847_3	19747_1_1	yes	yes	no	no	no	KM1_LIKE	K27M H3.1	1	0	R206H	1	0	1	astro	30	0	0	astro II	NA	Medium	1	Large necrotic area
P144	F	8.03	9.93	1	5	intermediate	good	Tarceva	0	28803_4	19720_2_3	yes	yes	no	yes	yes	KM1_LIKE	K27M H3.3	1	0	wild-type	0	0	1	oligo	25	NI	0	oligo astro II	NA	Medium	0	None
P145	M	7.48	37.79	1	3	intermediate	good	Tarceva	0	28842_3	19720_2_4	yes	yes	no	no	no	KM1_LIKE	K27M H3.1	1	0	G328V	1	0	1	oligo	25	NI	0	astro III	oligo A	Medium	0	None
P148	M	9.81	0.82	1	3	intermediate	good	Other	NA	28837_4	19747_2_2	yes	yes	yes	no	yes	KM1_LIKE	K27M H3.3	1	0	wild-type	0	1	1	astro	30	NI	1	astro III	NA	Medium	0	None
P149	F	4.83	10.43	1	6	intermediate	good	Other	0	NA	NA	NA	NA	NA	NA	NA	NA	K27M H3.3	1	0	NA	NA	0	NA	NA	NA	NA	NA	DIPG	DIPG	NA	1	Large necrotic area
P152	M	6.5	11.86	1	NA	NA	NA	NA	NA	28836_1	19768_1_4	no	no	no	no	yes	KM1_LIKE	K27M H3.3	1	0	wild-type	0	1	1	oligo	40	NI	0	astro III	oligo B	Medium	NA	NA
P157	M	5.73	11.57	1	7	high	good	Tarceva	0	28843_2	19769_1_2	no	no	no	no	yes	KM1_LIKE	K27M H3.3	1	0	wild-type	0	1	NA	oligo	30	NA	0	oligo astro II	oligo astro A	Low	1	Large necrotic area
P158	M	6.54	NA	0	NA	NA	NA	NA	NA	NA	19769_1_3	NA	NA	NA	NA	NA	KM1_LIKE	K27M H3.3	1	0	wild-type	0	1	0	astro	50	1	1	astro IV	TGNM	Low	NA	NA
P160	M	6.58	16.36	1	0	standard	good	Other	NA	28847_1	19769_2_1	yes	no	no	no	no	KM1_LIKE	K27M H3.3	1	0	wild-type	0	0	NA	oligo	50	NA	0	astro II	oligo A	Low	NA	NA
P162	F	10.65	9.18	1	NA	NA	NA	NA	NA	28843_4	19769_2_4	no	no	no	yes	yes	KM1_LIKE	K27M H3.3	1	0	wild-type	0	0	1	oligo	40	NI	0	astro III	oligo astro B	Low	1	Ring CE
P163	F	6.64	14.04	0	3	intermediate	NA	NA	NA	NA	NA	NA	NA	NA	NA	NA	NA	K27M H3.3	1	0	NA	NA	0	NA	oligo	15	NA	NA	astro II	oligo A	Low	0	None
P170	F	7.4	7.82	1	NA	NA	NA	NA	NA	NA	NA	NA	NA	NA	NA	NA	NA	K27M H3.3	1	0	NA	NA	0	1	astro	30	0	0	astro II	NA	Low	NA	NA
P171	F	6.49	14.61	1	0	standard	good	Other	NA	NA	19750_2_3	NA	NA	NA	NA	NA	KM1_LIKE	K27M H3.3	1	0	wild-type	0	0	1	oligo	15	NI	NA	astro II	oligo A	Low	0	None
P173	F	5.69	14	1	3	intermediate	good	Tarceva	NA	28843_1	19751_1_1	yes	no	no	yes	yes	KM1_LIKE	K27M H3.3	1	0	wild-type	0	0	NA	oligo	25	NA	0	astro III	oligo B	Medium	1	Ring CE
P174	M	3.7	9.39	1	11	high	poor	Radiotherapy	NA	28847_2	19751_1_2	yes	yes	no	no	no	KM2_LIKE	K27M H3.1	1	0	wild-type	0	0	1	astro	30	0	0	astro IV	GBM	Medium	1	Large necrotic area
P175	F	5.21	19.46	1	3	intermediate	good	Tarceva	NA	NA	NA	NA	NA	NA	NA	NA	NA	K27M H3.1	NA	NA	NA	NA	NA	NA	NA	NA	NA	NA	NA	NA	NA	1	Nodular CE
P176	F	6.75	6.89	1	3	intermediate	good	Other	1	NA	NA	NA	NA	NA	NA	NA	NA	K27M H3.3	NA	NA	NA	NA	NA	NA	NA	NA	NA	NA	NA	NA	NA	1	Ring CE
P177	F	5.8	7.93	1	1	intermediate	good	Tarceva	0	NA	NA	NA	NA	NA	NA	NA	NA	wild-type	NA	NA	NA	NA	NA	NA	NA	NA	NA	NA	NA	NA	NA	1	Nodular CE
P178	F	6.18	7.68	0	-1	standard	poor	Other	NA	NA	NA	NA	NA	NA	NA	NA	NA	K27M H3.3	NA	NA	NA	NA	NA	NA	NA	NA	NA	NA	NA	NA	NA	1	Nodular CE
P179	F	3.47	8.79	0	7	high	poor	Temodal	NA	NA	NA	NA	NA	NA	NA	NA	NA	K27M H3.1	NA	NA	NA	NA	NA	NA	NA	NA	NA	NA	NA	NA	NA	1	Large necrotic area
P180	F	15.29	9.5	0	10	high	good	Radiotherapy	NA	NA	NA	NA	NA	NA	NA	NA	NA	K27I H3.3	0	0	NA	NA	NA	NA	NA	20	NA	NA	astro II	DIPG	Low	1	Large necrotic area
P181	M	7.09	4.54	0	6	intermediate	poor	Tarceva	NA	NA	NA	NA	NA	NA	NA	NA	NA	K27M H3.3	NA	NA	NA	NA	NA	NA	NA	NA	NA	NA	NA	NA	NA	1	Ring CE
P182	F	9.74	12.75	1	5	intermediate	poor	Radiotherapy	NA	NA	NA	NA	NA	NA	NA	NA	NA	K27M H3.3	NA	NA	NA	NA	NA	NA	NA	NA	NA	NA	NA	NA	NA	0	None
P183	M	4.18	12.04	0	NA	NA	NA	NA	NA	NA	NA	NA	NA	NA	NA	NA	NA	K27M H3.1	NA	NA	NA	NA	NA	NA	NA	NA	NA	NA	NA	NA	NA	1	Ring CE
P184	M	11.79	14.36	0	2	intermediate	poor	Other	1	NA	NA	NA	NA	NA	NA	NA	NA	K27M H3.3	NA	NA	NA	NA	NA	NA	NA	NA	NA	NA	NA	NA	NA	0	None
P185	M	4.59	8.36	0	4	intermediate	poor	Tarceva	NA	NA	NA	NA	NA	NA	NA	NA	NA	K27M H3.1	NA	NA	NA	NA	NA	NA	NA	NA	NA	NA	NA	NA	NA	1	Large necrotic area
P186	F	5.13	6	1	7	high	good	Tarceva	NA	NA	NA	NA	NA	NA	NA	NA	NA	K27M H3.3	NA	NA	NA	NA	NA	NA	NA	NA	NA	NA	NA	NA	NA	1	Large necrotic area
P187	M	25.33	13.57	1	3	intermediate	good	Temodal	1	NA	NA	NA	NA	NA	NA	NA	NA	K27M H3.3	NA	NA	NA	NA	NA	NA	NA	NA	NA	NA	NA	NA	NA	0	None
P188	F	4.31	5.11	1	7	high	good	Other	1	NA	NA	NA	NA	NA	NA	NA	NA	wild-type	0	1	NA	NA	1	NA	NA	60	NA	NA	astro IV	TGNM	Low	1	Large necrotic area
P189	F	4.07	4.5	0	2	intermediate	good	Tarceva	NA	NA	NA	NA	NA	NA	NA	NA	NA	K27M H3.3	NA	NA	NA	NA	NA	NA	NA	NA	NA	NA	NA	NA	NA	0	None
P190	F	5.77	8.79	1	7	high	poor	Other	0	NA	NA	NA	NA	NA	NA	NA	NA	K27M H3.3	NA	NA	NA	NA	NA	NA	NA	NA	NA	NA	NA	NA	NA	1	Ring CE
P191	F	4.4	9.32	0	2	intermediate	good	Other	NA	NA	NA	NA	NA	NA	NA	NA	NA	K27M H3.1	NA	NA	wild-type	0	NA	NA	NA	NA	NA	NA	NA	NA	NA	0	None
P192	F	5.23	6.89	0	7	high	good	Other	NA	NA	NA	NA	NA	NA	NA	NA	NA	K27M H3.1	NA	NA	NA	NA	NA	NA	NA	NA	NA	NA	NA	NA	NA	1	Large necrotic area
P193	F	11.55	3.14	1	7	high	poor	Tarceva	1	NA	NA	NA	NA	NA	NA	NA	NA	K27M H3.3	NA	NA	NA	NA	NA	NA	NA	NA	NA	NA	NA	NA	NA	1	Large necrotic area
P194	M	3.99	17.14	0	1	intermediate	NA	NA	1	NA	NA	NA	NA	NA	NA	NA	NA	K27M H3.1	NA	NA	NA	NA	NA	NA	NA	NA	NA	NA	NA	NA	NA	1	Nodular CE
P195	M	10.2	8.54	0	0	standard	good	Tarceva	NA	NA	NA	NA	NA	NA	NA	NA	NA	K27M H3.3	NA	NA	NA	NA	NA	NA	NA	NA	NA	NA	NA	NA	NA	0	None
P198	M	4.67	8.29	NA	NA	NA	NA	NA	NA	NA	NA	NA	NA	NA	NA	NA	NA	K27M H3.2	1	0	NA	NA	0	NA	NA	50	NA	NA	oligo astro III	oligoastro B	Medium	NA	NA

Suppl. Table S2: **PCR primers**

Primer name	Primer sequence (5' - 3')
HIST1H3B_F	GGGCAGGAGCCTCTCTTAAT
HIST1H3B _R	ACCAAGTAGGCCTCACAAGC
Hist1H3C_F	CTCATTGCAAATGGCTCGTA
Hist1H3C_R	CATGATGGTGACGCGTTTAG
Hist2H3C_F	TGGCCCGTACTAAGCAGACT
Hist2H3C_R	AGCGGTACAGCTTCTTCCAA
H3F3A_V2_F	GTGATCGTGGCAGGAAAAGT
H3F3A_V2_R	GGTTTCTTCACCCCTCCAGT
H3F3A_V1_F	GATTTTGGGTAGACGTAATCTTC
H3F3A_V1_R	TTTCCTGTTATCCATCTTTTTGTT
ACVR1-gen-R206-F	TCAGGAAGTGGCTCTGGTCT
ACVR1-gen-R206-R	CAAGTTCAGGTGCTCCAACA
ACVR1-gen-R258-F	AAGGCAGGTATGGTGAGGTG
ACVR1-gen-R258-R	CCAAAACGGAGAGAGCAAAG
ACVR1-gen-G328-F	GCCTTCGAATAGTGCTGTCC
ACVR1-gen-G328-R	AACATGTTGTGGGGGAGAGA
ACVR1-gen-G356-F	AGGTAGCCTAGCGGGAGAAG
ACVR1-gen-G356-R	CGGCCAGATCGATTTACAAG

Suppl. Table S3: List of differentially expressed genes.

Official Gene Symbol	H3.1/ H3.3 ratio	Adjusted *p*-value
**HOXD8**	**18.17**	**1.56E-07**
**TLX2**	**11.98**	**9.19E-05**
ANGPTL4	5.89	1.18E-02
ADM	5.85	1.18E-02
SLC30A3	5.83	3.41E-02
**BNC1**	**4.31**	**1.99E-07**
TBX1	3.73	2.48E-02
NGFR	3.64	1.97E-02
IFITM4P	3.61	2.48E-02
IFITM2	3.58	1.18E-02
IFITM3	3.55	2.42E-02
EGFLAM	2.58	1.89E-02
FRZB	2.46	2.48E-02
**FGF11**	**2.40**	**1.49E-03**
**IRX4**	**2.37**	**2.21E-03**
LDHA	2.31	2.06E-02
**PMAIP1**	**2.31**	**4.06E-03**
MRAP	2.25	2.42E-02
CD14	2.21	3.30E-02
**LSP1**	**2.17**	**9.97E-03**
ITGA6	2.13	3.99E-02
SLC40A1	2.06	3.81E-02
GPR116	2.05	3.33E-02
ITPKB	2.05	2.96E-02
PYGL	2.05	3.47E-02
FOLR2	2.03	3.75E-02
CDKN1A	2.01	4.04E-02
**PRRX1**	**2.00**	**3.20E-03**
ERO1L	2.00	1.18E-02
SLC16A4	1.99	1.39E-02
**DDB2**	**1.97**	**2.89E-03**
C1orf54	1.93	1.30E-02
STAB1	1.92	1.50E-02
**HK2**	**1.92**	**7.94E-03**
FADS2	1.90	4.49E-02
SHC1	1.89	2.25E-02
LRRC17	1.82	4.04E-02
PLXND1	1.80	3.54E-02
TNS1	1.79	2.48E-02
CACNA2D4	1.79	3.77E-02

ITGA1	1.79	2.96E-02
EPAS1	1.76	3.30E-02
**RGL3**	**1.76**	**1.49E-03**
RAB7L1	1.75	1.93E-02
ANO7	1.73	1.67E-02
PPP1R3B	1.71	3.87E-02
CLEC1A	1.71	1.63E-02
PDK1	1.67	4.20E-02
SLC27A3	1.66	4.24E-02
RHOB	1.65	2.48E-02
NOSTRIN	1.65	3.33E-02
INSIG2	1.63	4.04E-02
MSC	1.63	1.46E-02
FRMD6	1.61	3.45E-02
**FDXR**	**1.60**	**4.06E-03**
EDA2R	1.60	2.17E-02
MYO10	1.58	2.86E-02
**GABPB2**	**1.58**	**7.94E-03**
**ABHD1**	**1.57**	**7.08E-03**
FAM57A	1.54	3.86E-02
PDIA6	1.53	3.30E-02
AEN	1.48	2.96E-02
TPR	1.47	1.93E-02
FLVCR2	1.47	1.67E-02
ZNF366	1.47	2.96E-02
**AP1S3**	**1.44**	**9.74E-03**
DERL2	1.42	3.47E-02
ITGA10	1.41	4.13E-02
ELP5	1.40	2.48E-02
DSP	1.38	3.27E-02
EGLN1	1.37	2.24E-02
STARD13	1.36	3.01E-02
TOR1AIP2	1.31	2.61E-02
BLZF1	1.30	1.74E-02
DMBX1	1.27	2.96E-02
MSLN	1.23	3.27E-02
ANKFY1	1.22	2.68E-02
IL6R	1.21	2.48E-02
ALX1	1.18	4.12E-02
GRIK3	0.82	4.49E-02
CMPK1	0.81	4.37E-02
XCR1	0.77	3.45E-02
HTT	0.76	3.01E-02
MEAF6	0.74	2.18E-02
BCORL1	0.74	2.96E-02
KCNA3	0.74	1.47E-02

CROCC	0.73	2.06E-02
ZP3	0.73	4.65E-02
NUDT10	0.73	2.48E-02
RCC2	0.71	3.07E-02
ATAD3A	0.70	4.04E-02
ZNRF1	0.70	4.32E-02
MGAT5B	0.70	4.04E-02
CDK5R1	0.70	2.85E-02
CCDC158	0.69	3.30E-02
**TMEM240**	**0.68**	**4.57E-03**
KCNG3	0.68	1.18E-02
RAB9B	0.67	3.30E-02
PCDH11X	0.67	3.63E-02
TCEB2	0.67	1.89E-02
HPCA	0.66	3.87E-02
NUDT11	0.66	4.04E-02
TMEM132B	0.66	2.96E-02
ELAVL3	0.66	4.93E-02
DES	0.64	3.33E-02
PRDM8	0.64	4.37E-02
AMER1	0.63	4.49E-02
TLE4	0.63	3.45E-02
ZSCAN1	0.62	2.48E-02
WFIKKN1	0.61	4.37E-02
CTNNBIP1	0.61	2.48E-02
**MEF2B**	**0.60**	**3.20E-03**
CBX8	0.60	4.98E-02
PRKCZ	0.59	2.48E-02
PCYT1B	0.59	2.82E-02
TRPC3	0.59	4.04E-02
CACNA1A	0.58	1.10E-02
PPM1E	0.58	2.48E-02
SBK1	0.58	1.38E-02
GLT1D1	0.58	2.96E-02
FAM131B	0.58	3.09E-02
PCSK1N	0.57	2.68E-02
C2orf74	0.57	4.37E-02
**DLG3**	**0.57**	**3.20E-03**
ZCCHC12	0.57	2.93E-02
WFDC2	0.56	1.04E-02
FAM184B	0.54	2.48E-02
SYT14	0.54	1.66E-02
KCNMB2	0.54	2.48E-02
SHISA9	0.53	4.04E-02
LRRC73	0.52	3.54E-02
ASIC1	0.52	2.42E-02

FBLL1	0.52	3.01E-02
**ADAMTS17**	**0.52**	**7.90E-04**
DLGAP3	0.50	3.54E-02
IRX5	0.49	2.06E-02
CAMTA1	0.49	2.85E-02
**CAMK2N2**	**0.48**	**1.49E-03**
CACNG1	0.47	3.54E-02
RPRM	0.47	4.12E-02
RIMS2	0.46	1.93E-02
PDGFRA	0.43	2.25E-02
HS6ST2	0.43	4.61E-02
CA10	0.42	2.42E-02
PCDH11Y	0.42	3.41E-02
TMEFF2	0.42	3.54E-02
ATCAY	0.41	1.18E-02
SLC35F3	0.40	2.48E-02
CHRDL1	0.38	3.69E-02
WNT6	0.38	2.25E-02
CNGA3	0.37	1.13E-02
**CMYA5**	**0.37**	**1.32E-03**
KLRC1	0.33	4.84E-02
SPHKAP	0.32	3.38E-02
NPM2	0.29	3.27E-02
**TSPYL5**	**0.29**	**4.03E-03**
MGARP	0.28	3.54E-02
MOXD1	0.27	2.96E-02
ARHGEF5	0.27	1.93E-02
**MAB21L1**	**0.24**	**1.32E-03**
